# Hydride oxidation from a titanium–aluminum bimetallic complex: insertion, thermal and electrochemical reactivity†
†Electronic supplementary information (ESI) available: Experimental details, NMR, IR, and UV-vis spectra, cyclic voltammetry, computational, and EI-MS data, crystallographic details and files in CIF format. CCDC 1530360–1530369. For ESI and crystallographic data in CIF or other electronic format see DOI: 10.1039/c7sc01835e


**DOI:** 10.1039/c7sc01835e

**Published:** 2017-05-31

**Authors:** Alexandra C. Brown, Alison B. Altman, Trevor D. Lohrey, Stephan Hohloch, John Arnold

**Affiliations:** a Department of Chemistry , University of California , Berkeley , California 94720 , USA . Email: arnold@berkeley.edu; b Chemical Sciences Division , Lawrence Berkeley National Laboratory , Berkeley , California 94720 , USA

## Abstract

We report the synthesis and reactivity of paramagnetic heterometallic complexes containing a Ti(iii)-μ-H-Al(iii) moiety. Combining different stoichiometries amounts of Cp_2_TiCl and KH_3_AlC(TMS)_3_ (Cp = cyclopentadienyl, TMS = trimethylsilyl) resulted in the formation of either bimetallic Cp_2_Ti(μ-H)_2_(H)AlC(TMS)_3_ (**2**) or trimetallic (Cp_2_Ti)_2_(μ-H)_3_(H)AlC(TMS)_3_ (**3**) *via* salt metathesis pathways. While these complexes were indefinitely stable at room temperature, the bridging hydrides were readily activated upon exposure to heteroallenes, heating, or electrochemical oxidation. In each case, formal hydride oxidation occurred, but the isolated product maintained the +3 oxidation state at both metal centers. The nature of this reactivity was explored using deuterium labelling experiments and Density Functional Theory (DFT) calculations. It was found that while C–H activation from the Ti(iii) bimetallic may occur through a σ-bond metathesis pathway, chemical oxidation to Ti(iv) promotes bimolecular reductive elimination of dihydrogen to form a Ti(iii) product.

## Abstract 2

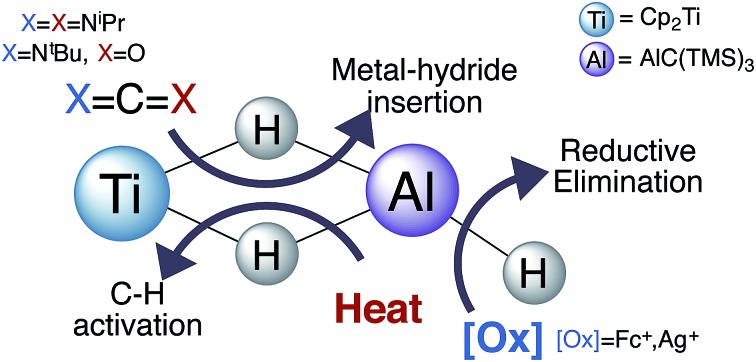
We report the synthesis and reactivity of paramagnetic heterometallic complexes containing a Ti(iii)-μ-H-Al(iii) moiety.

## Introduction

Aluminum hydrides are important for a wide range of applications, from organic synthesis to hydrogen storage.^1^–^7^ These species are often poorly defined extended solids with a variety of phase mixtures and defects.^8^,^9^ This complexity obscures the mechanistic details of the varied transformations these materials mediate. In contrast, molecular systems offer well-defined metal sites that allow for in-depth study of the reactivity of aluminum-hydride bonds. Recent synthetic work^10^–^12^ and a spectroscopic study^13^ provide evidence that specific ligand frameworks support reductive elimination of aluminum hydrides under mild conditions to yield aluminum(i) compounds. We were interested in exploring the relevance of this chemistry to titanium–aluminum bimetallics, as aluminum materials doped with titanium are used for hydrogen storage, and the redox processes behind the repeated storage and discharge of hydrogen from such materials remain not well understood.^14^–^20^

**Fig. 1:**
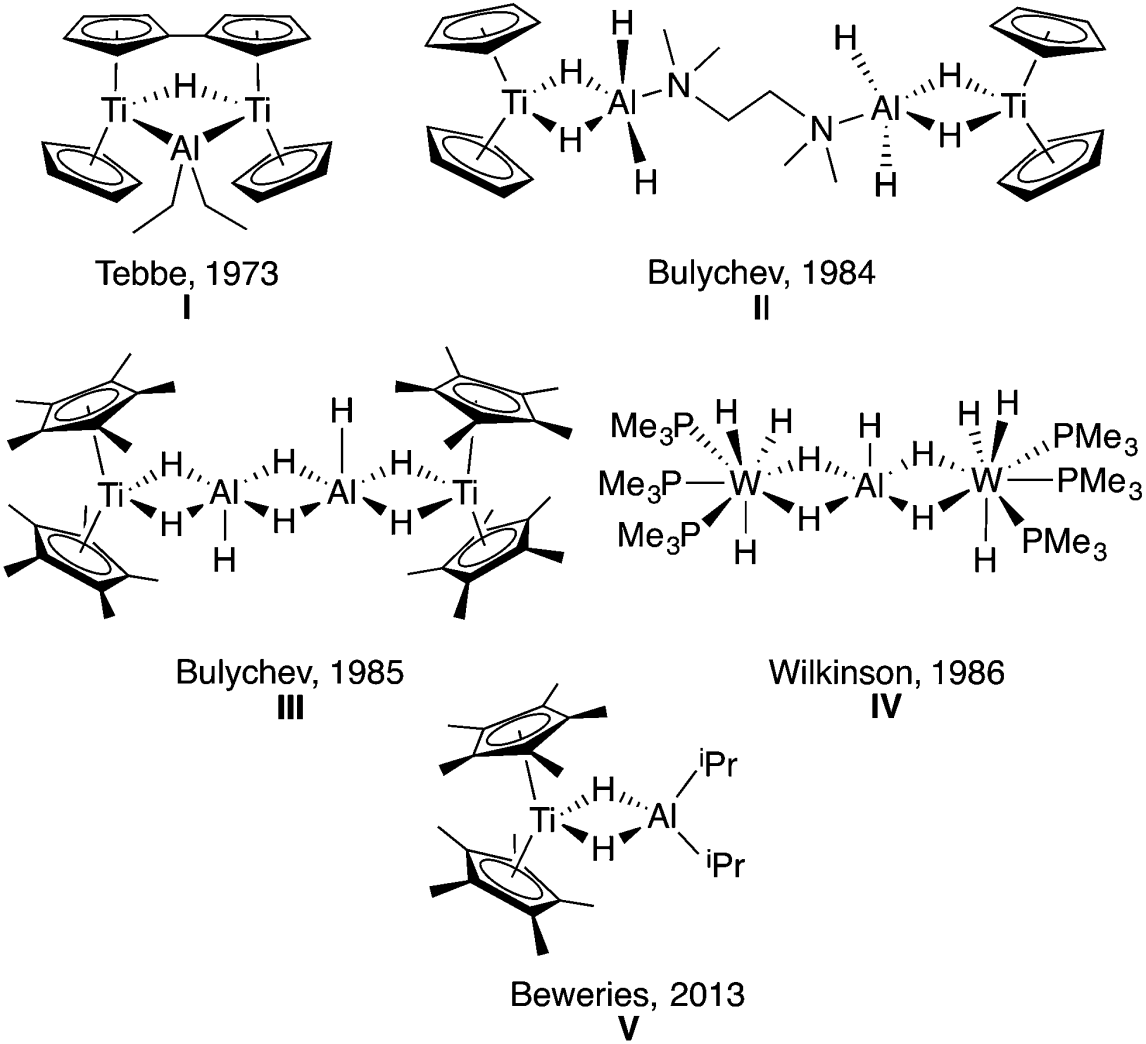
Previously synthesized transition metal aluminum hydride heterometallics **I**,^39^**II**,^31^**III**,^29^**IV**^22^ and **V**^41^ showing the various motifs that have been isolated.

Previous work by Wilkinson and coworkers,^21^–^24^ Bulychev and coworkers,^25^–^36^ Stephan and coworkers,^37^,^38^ and Tebbe and coworkers^39^,^40^ found that the reaction of transition metal halides and LiAlH_4_ formed multimetallic structures containing the TM-μ-H-Al motif (Fig. 1). Bulychev and coworkers^25^–^34^ were able to synthesize a variety of titanium–aluminum heterometallics. They explored stoichiometric protonolysis reactivity to form alkoxide and amide complexes^32^,^33^ and showed the promise of these titanium–aluminum hydrides, alkoxides, amides and halides for the catalytic hydrogenation and isomerization of olefins.^26^,^27^,^29^,^33^,^34^

As outlined in the recent comprehensive review by Crimmin and coworkers,^42^ while the structural parameters, protonolysis, and olefin reactivity of aluminum hydride heterobimetallics are well established, there are opportunities to develop other classes of reactions. To this end, there have been recent reports of *in situ* generated titanium aluminum bimetallics for catalytic dehydrogenation of hydrazine borane (**V**)^41^ and catalytic hydroalumination of alkynes.^43^

These species offer promising new avenues in catalysis, and to better understand the breadth and opportunities in titanium–aluminum bimetallic reactivity, it is necessary to conduct detailed study of isolated heterobimetallics under a variety of conditions. To form these discrete bimetallics, with thermal stability and potential redox activity, we have employed a bulky alanate stabilized by a tris(TMS)methyl ligand. This is the only ligand we are aware of known to support an aluminum(iii) alanate as well as a low-valent aluminum(i) species; such redox activity is required for the reversible storage of dihydrogen.^44^–^51^ Here, we report that a titanium(iii)–aluminum bimetallic complex incorporating this ligand was found to be indefinitely stable at room temperature under inert atmosphere, but the bridging hydrides were readily oxidized. Utilizing this bimetallic, we have further investigated thermal hydrogen evolution and have developed of new classes of reactivity for titanium–aluminum hydrides: reduction of heteroallenes and reductive elimination of dihydrogen upon electrochemical oxidation.

## Results and discussion

**Scheme 1:**
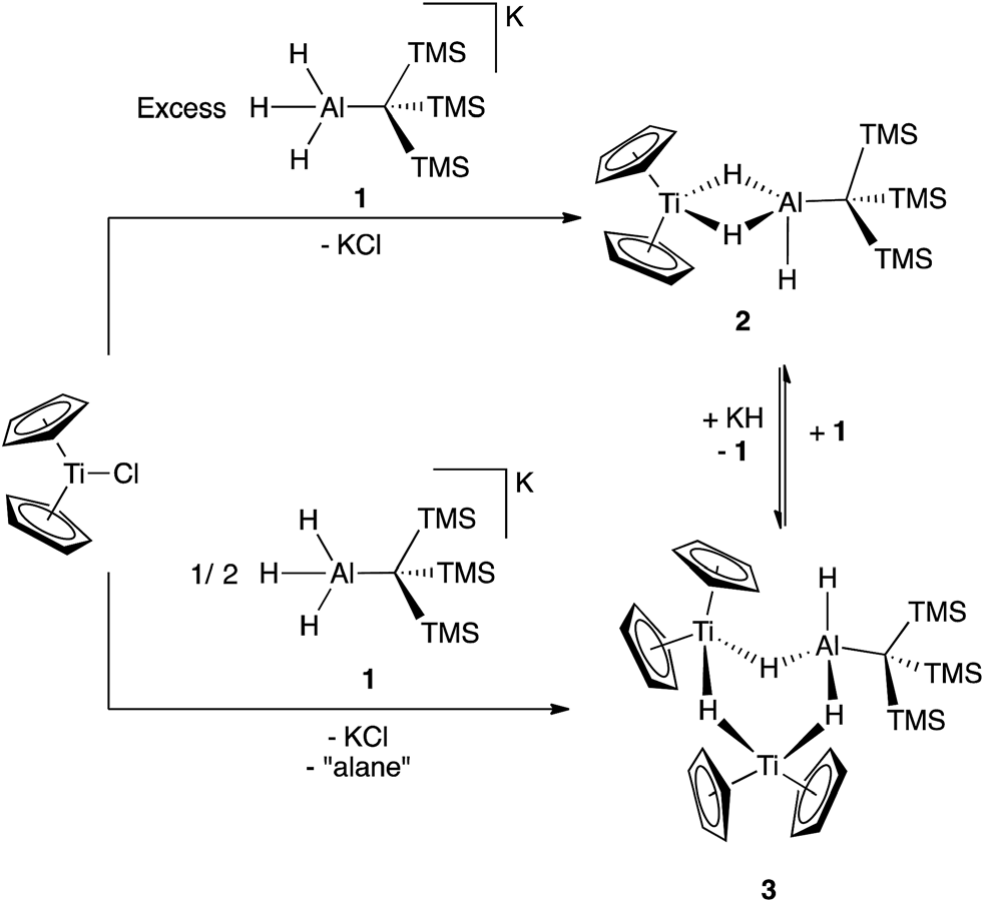
Formation and interconversion of bimetallic **2** and trimetallic **3**.

The reaction of Cp_2_TiCl with KH_3_AlC(TMS)_3_ (**1**) in 1 : 1 ratios generated the paramagnetic bimetallic compound Cp_2_Ti(μ-H)_2_(H)AlC(TMS)_3_ (**2**) *via* salt metathesis. Upon addition of **1** to Cp_2_TiCl in diethyl ether, there was an instantaneous color change from green to purple. The ^1^H NMR spectrum of the crude material revealed two broad resonances at 0.35 ppm and 0.33 ppm; the relative intensities were found to be dependent on the initial ratios of **1** to Cp_2_TiCl. While these complexes are paramagnetic, the presence of a broad resonance between 0.3 and 0.5 ppm is diagnostic of the –C(TMS)_3_ protons. Through optimization of synthetic procedures, we found that compound **2** could be formed selectively by reaction of Cp_2_TiCl with **1** in a 1 : 2 ratio in 83% yield, (see ESI†) while (Cp_2_Ti)_2_(μ-H)_3_(H)AlC(TMS)_3_ (**3**) could be formed from a 2 : 1 ratio of Cp_2_TiCl with **1** in 32% yield (Scheme 1).

**Fig. 2:**
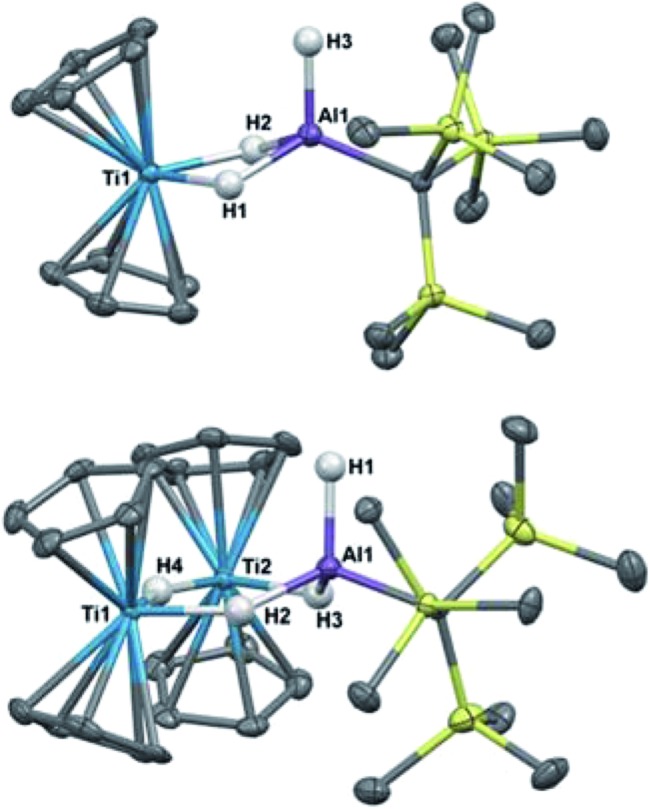
Crystallographically determined structures of **2** (top) and **3** (bottom). Thermal ellipsoids are shown at the 50% probability level. C–H hydrogens are omitted for clarity. Metal hydrides were located in the Fourier difference map and their positions were refined. Ti(1)–Al(1) distance in **2**: 2.7773(8) Å. Metal–metal distances in **3**: Ti(1)–Al(1): 3.286(2) Å, Ti(2)–Al(1): 3.264(2) Å, Ti(1)–Ti(2): 3.761(1) Å.

Crystallographic characterization of **2** and **3** (Fig. 2) confirmed formation of two heterometallic complexes incorporating a Ti(iii)-μ-H-AlH motif. The bimetallic **2** contains one titanium(iii) atom and one aluminum atom at a distance of 2.7773(8) Å. This separation is very similar to those reported by Bulychev (2.788 Å,^31^ 2.750(3) Å 29) and while it is less than the sum of the covalent radii^52^ (2.81(9) Å), there is no further indication of metal–metal bonding. In comparison, the trimetallic **3** contains two titanium(iii) atoms and one aluminum atom as part of a six-membered ring, with the metals bridged by hydrides. The Ti–Al separations (3.286(2) Å and 3.264(2) Å) and the Ti–Ti separation (3.761(1) Å) are within the sum of the van der Waals radii^53^ (Ti/Al: 4.25 Å, Ti/Ti: 4.30 Å), but the metals are much farther apart than in **2** and any metal–metal interaction, if present, must be much weaker. We confirmed the paramagnetism of **2** and **3** using the Evans NMR method;^54^,^55^ after diamagnetic correction^56^,^57^ the magnetic moment of **2** is 1.70(2) *μ*_B_ (calculated spin-only moment: 1.73 *μ*_B_), while the magnetic moment of the [Ti(iii)]_2_ unit in **3** is 1.66(2) *μ*_B_ (calculated spin-only moment for two independent d^1^ Ti(iii) centers: 2.83 *μ*_B_) indicative of antiferromagnetic coupling between the two Ti(iii) centers.

We investigated the behavior of **2** in solution, with the hypothesis that **2** might dissociate into [Cp_2_TiH] and H_2_AlC(TMS)_3_ in solution since evidence of such dissociation is reported in related zirconium systems.^58^–^60^ However, Cp_2_TiH is known to be extremely unstable in solution at room temperature.^61^ If the bimetallic were in equilibrium with its dissociated monometallic components, we expect that decomposition of transiently formed Cp_2_TiH in solution would drive the dissociation equilibrium towards full decomposition of **2**. The observed stability of **2** and **3** indicates that the solid-state structures are representative of the species present in solution.

Though **2** and **3** are stable in solution, they interconvert upon addition of additional reagents. Compound **2** is slowly converted to **3** upon addition of KH and **3** can be converted to **2** by the addition of **1** (Scheme 1). This explains the optimized stoichiometry; when a 2-fold excess of **1** is used, any **3** formed transiently is converted to **2**. Use of only 1/2 equivalent of **1** favors the formation of **3** over **2**, but the yield of **3** is limited because **1** also serves as a source of hydrides in this reaction. While alane products are predicted stoichometrically, no aluminum byproducts were isolated from these reactions.

### Insertion reactivity

With a high-yielding synthesis of a titanium–aluminum bimetallic, we had the opportunity to pursue novel reactivity. Addition of 1 atm of CO_2_ to **2** in solution resulted in a color change from purple to teal. While the FTIR spectrum of the crude product contained a formate stretch (see ESI†), no pure crystalline material could be isolated. We then turned to the reduction of other heteroallenes as analogues to CO_2_, where we met more success.

**Scheme 2:**
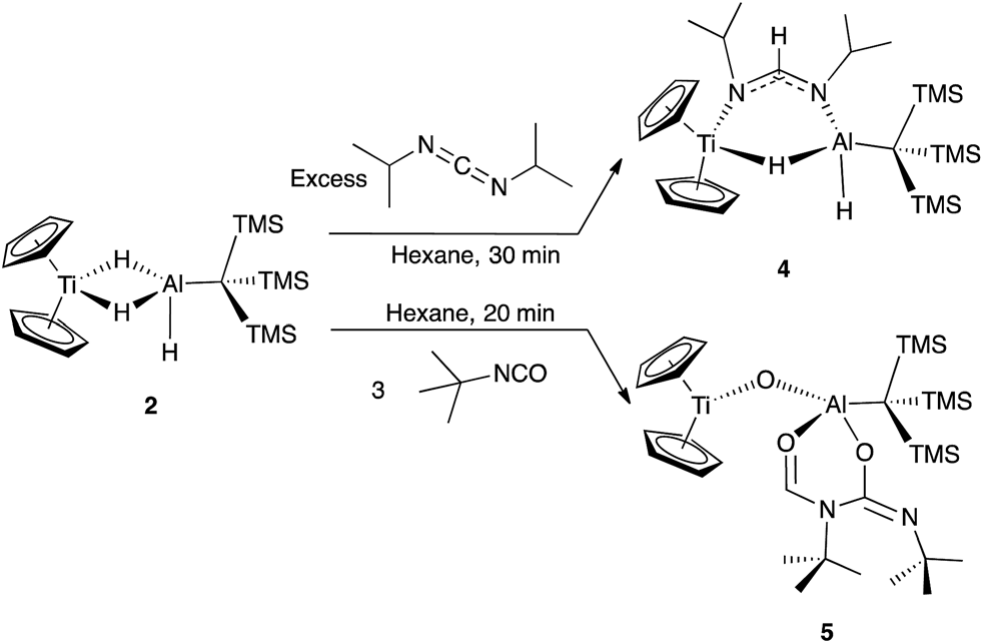
Organic substrate reduction reactivity of **2** with diisoproylcarbodiimide and *tert*-butyl isocyanate.

**Fig. 3:**
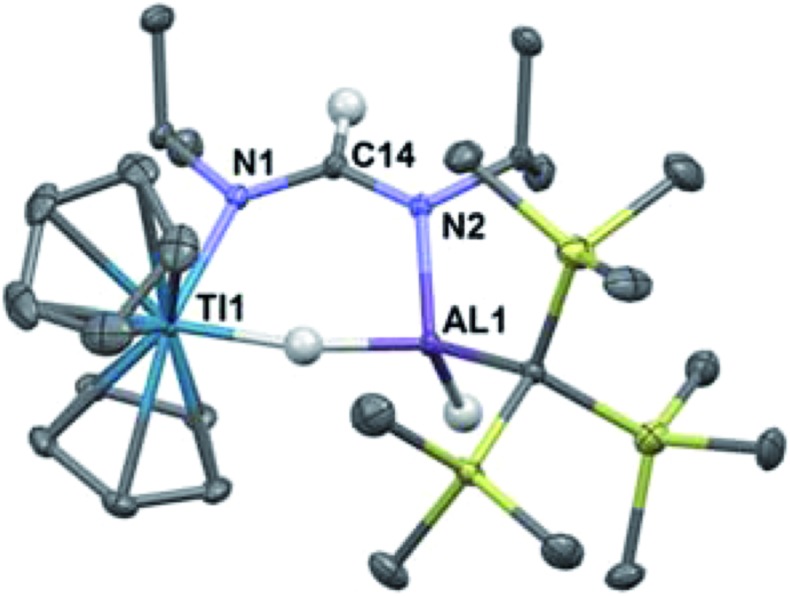
Crystallographically determined structure of **4**. Thermal ellipsoids are shown at the 50% probability level. Metal hydrides were located in the Fourier difference map and their positions were refined. C–H hydrogen atoms are omitted for clarity. Relevant distances and bond lengths: Ti(1)–Al(1) 3.300(1) Å, N(1)–C(14): 1.298(2) Å, N(2)–C(14): 1.331(2) Å, Ti(1)–N(1): 2.161(1) Å, Al(1)–N(2) 1.892(1) Å.

Reaction of **2** with an excess of diisopropylcarbodiimide led to the isolation of deep blue crystals of Cp_2_Ti(μ-H)(μ-(iPr)N(CH)N(iPr))(H)AlC(TMS)_3_ (**4**) (Scheme 2). Compound **4** was characterized crystallographically, and features an anionic formamidinate trapped between titanium and aluminum centers that are 3.300(1) Å apart (Fig. 3). The formamidinate is asymmetric (1.298(2) Å (N1–C14) and 1.331(2) Å (N2–C14)), which suggests that the anionic charge of the formamidinate is localized towards the aluminum center. Notably, use of one equivalent of carbodiimide gave incomplete conversion to **4**, while use of a large excess did not result in further reactivity. Similar insertions are possible with other carbodiimides of comparable steric bulk such as di(*p*-tolyl)carbodiimide (see ESI†).

To investigate if modulating the steric properties of the heteroallene could result in activation of multiple hydrides in the bimetallic, we studied reactivity of **2** with isocyanates. Addition of three equivalents of *tert*-butylisocyanate to **2** in hexane led to the precipitation of a bright green solid, which was crystallographically determined to be Cp_2_Ti(μ-O)Al(OCHN^*t*^BuC(N^*t*^BuO)(C(TMS)_3_)) (**5**) (Scheme 2). No products could be isolated using fewer equivalents of isocyanate.

**Fig. 4:**
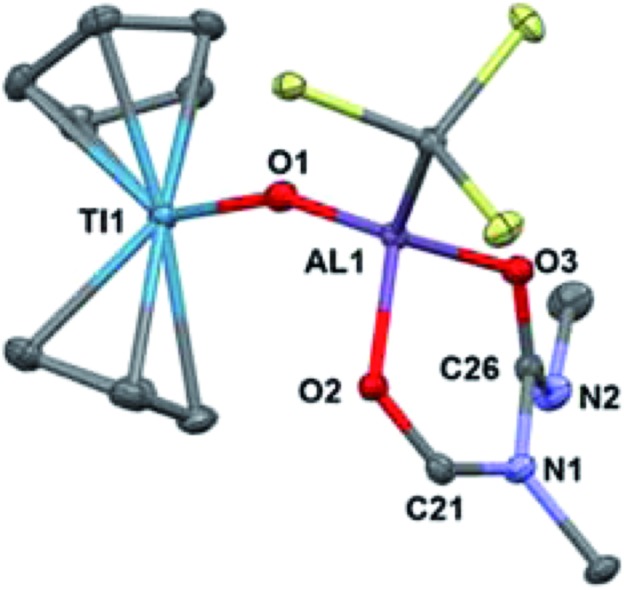
Crystallographically determined structure of **5**. Metal hydrides were located in the Fourier difference map and their positions were refined. Methyl groups and C–H hydrogens are omitted for clarity. Thermal ellipsoids are shown at the 50% probability level. Relevant bond lengths: Ti(1)–O(1): 1.838(2) Å, Al(1)–O(1): 1.705(2) Å, Al(1)–O(2): 1.845(2) Å, Al(1)–O(3): 1.795(2) Å, O(2)–C(21): 1.268(3) Å, C(21)–N(1): 1.322(3) Å, N(1)–C(26): 1.478(3) Å, C(26)–N(2): 1.262(3) Å, C(26)–O(3): 1.310(3) Å.

Complex **5** features a bridging titanium–aluminum oxo moiety in addition to a six-membered ring containing the aluminum center and two coupled isocyanates (Fig. 4). Such isocyanate insertion products are uncommon: a related example was recently reported with a boron-based Lewis acid.^62^ Despite isocyanate being a weak oxidizing agent, the product retains the Ti(iii) center; one hydride reduced the backbone carbon and two hydrides were transferred to an isocyanate fragment which was presumably the source of the oxo moiety, however no other products could be identified. Bulky isocyanates such as bis(2,6-diisopropylphenyl)isocyanate did not lead to isolable products.

While future work will expand substrate scope and explore the mechanism for formation of **5**, the reactivity observed shows that **2** promotes formal hydride oxidation and reduction of a range of heteroallenes. The differences between **4** and **5** suggest that decreased steric pressure promotes coupling of the organic substrates. We hypothesize that similar effects are relevant to CO_2_ reduction. Overall, this reactivity suggests that bimetallics could be useful for elucidating intermediates in aluminum hydride reductions.

### Thermal reactivity

**Scheme 3:**
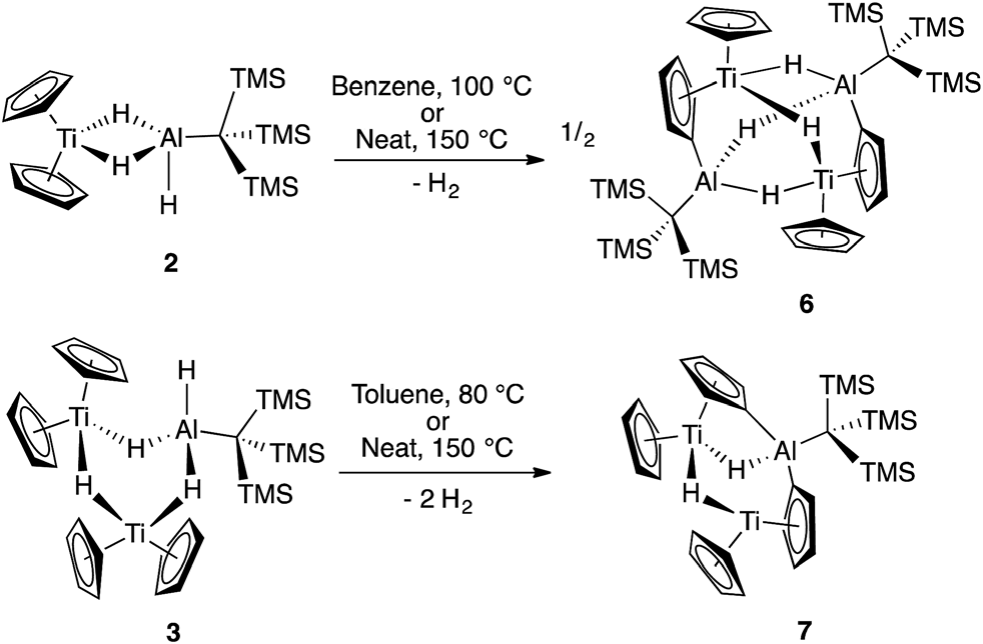
Thermal reactivity of **2** and **3** leading to **6** and **7**.

**Fig. 5:**
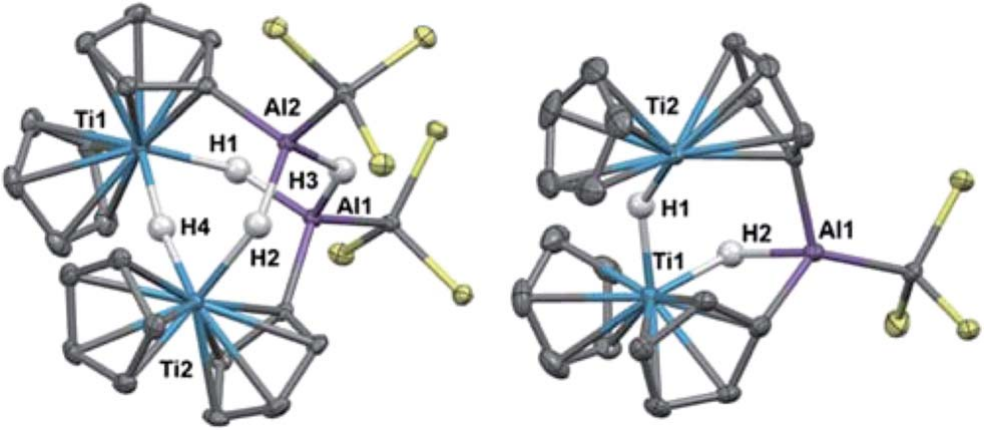
Crystallographically determined structures of **6** (left) and **7** (right). Methyl groups and C–H hydrogens are omitted for clarity. Thermal ellipsoids are shown at the 50% probability level. Metal hydrides were located in the Fourier difference map and their positions were refined. Metal–metal distances in **6**: Ti(1)–Al(2) 3.6235(8) Å, Ti(2)–Al(1): 3.6218(7) Å, Ti(1)–Ti(2): 3.6930(6) Å, Al(1)–Al(2): 3.2214(9) Å. Metal–metal distances in **7**: Ti(1)–Al(1): 2.976(1) Å, Ti(2)–Al(1): 3.594(1) Å, Ti(1)–Ti(2): 3.3074(7) Å.

Previous work with titanium–aluminum bimetallics focused on thermal decomposition *via* hydrogen elimination and C–H bond activation. To determine if **2** and **3** could undergo similar reactivity, we heated each in solution and as neat solids. Monitoring the solution-state reactions by ^1^H NMR spectroscopy revealed that dihydrogen formed at around 100 °C (**2**) and 80 °C (**3**), concomitant with color changes from purple to blue and from purple to brown for **2** and **3**, respectively (Scheme 3). Both products were crystallographically characterized and shown to be ((Cp)(C_5_H_4_)Ti(μ-H)_2_Al(C(TMS)_3_))_2_ (**6**) and ((Cp)(C_5_H_4_)Ti)_2_(μ-H)_2_Al(C(TMS)_3_) (**7**) (Fig. 5). In both **2** and **3**, heat induced activation of Cp C–H bonds and loss of one bridging hydride generated dihydrogen and new heterometallic complexes with C–Al bonds and η^1^:η^5^ coordinated activated Cp rings. This mode of reactivity can be compared with previous Ti–Al bimetallic complexes in which Cp C–H activation was observed at or below room temperature.^27^,^31^

**Fig. 6:**
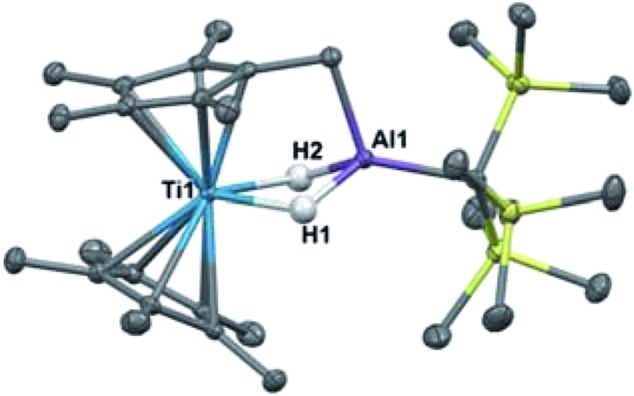
Crystallographically determined structure of **8**. Metal hydrides were located in the Fourier difference map and their positions were refined. Thermal ellipsoids are shown at the 50% probability level. C–H hydrogen atoms are omitted for clarity. Relevant distances and bond lengths: Ti(1)–Al(1) 2.8133(7) Å, Al(1)–C(11): 1.978(2).

**Scheme 4:**
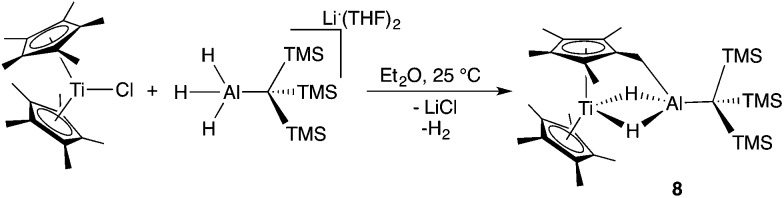
Synthesis of **8**.

Bulychev and coworkers found that substituting pentamethylcyclopentadienyl (Cp*) rings for Cp inhibited C–H activation in [AlH_4_]^–^ based Ti–Al bimetallics.^29^,^30^ We observed that the reaction of Cp*_2_TiCl with one equivalent of Li(THF)_2_H_3_AlC(TMS)_3_ in hexane or diethyl ether was significantly slower than the Cp analogue; a color change from dark blue to green was observed over several days at room temperature. X-ray structure determination of the green crystals (Fig. 6) showed that C–H activation had occurred to form (Cp*)(C_5_Me_4_CH_2_)Ti(μ-H)_2_Al(C(TMS)_3_) (**8**), presumably with the concomitant elimination of one equivalent of dihydrogen (Scheme 4). Complex **8** is unaffected by heating in the presence of 1 atm of H_2_ at 80 °C for 16 hours, which indicates that the elimination of dihydrogen is irreversible. The structure is reminiscent of tuck-in complexes that have been observed in metallocene derivatives across the periodic table.^63^,^64^

**Fig. 7:**
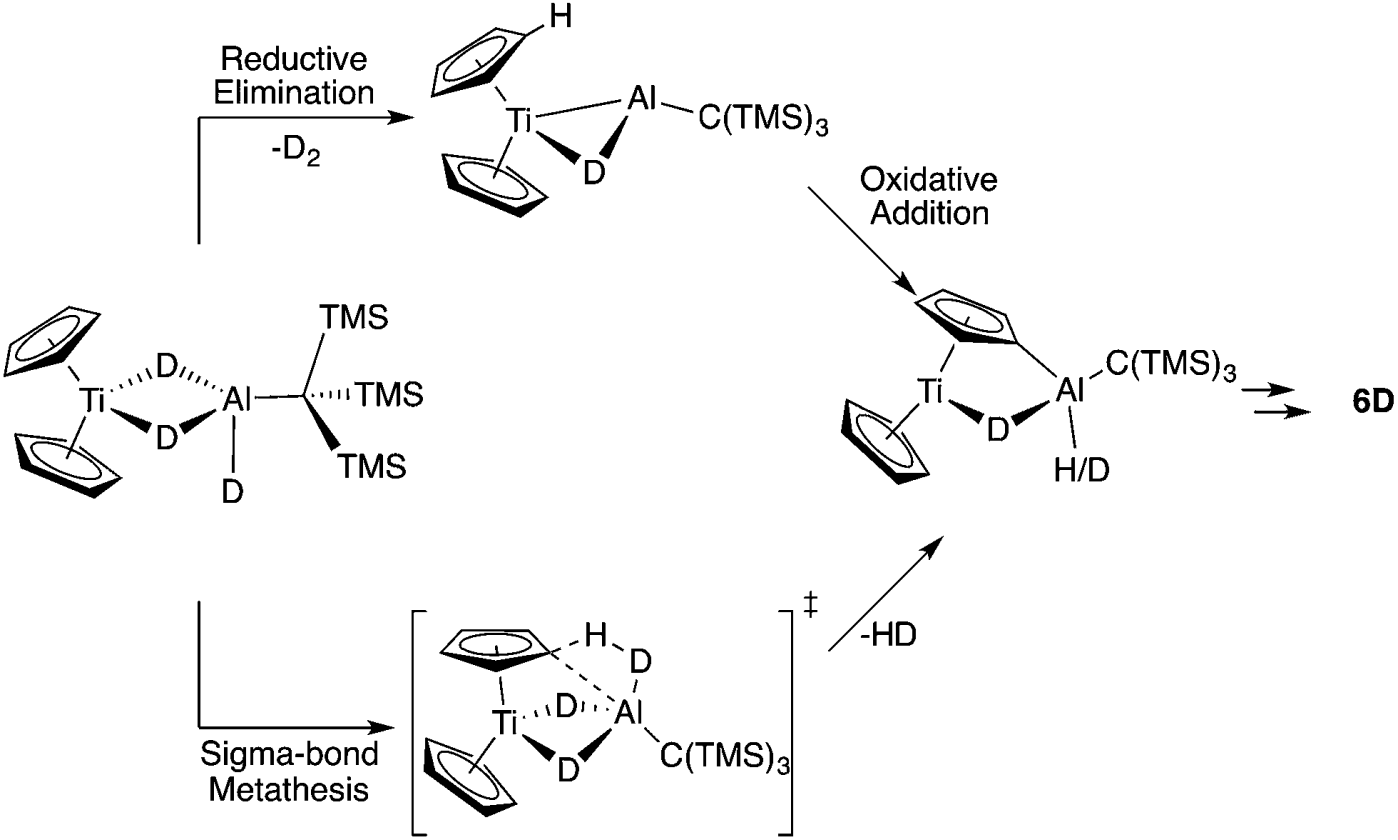
Possible model mechanisms for the conversion of **2** to **6** showing the expected results from deuterium labelling. The reaction may take place through a unimolecular or bimolecular pathways but for simplicity the unimolecular model has been shown.

We considered two mechanistic pathways for the formation of **6**, **7**, and **8**. The first – proposed by Bulychev and coworkers^27^ – involves reductive elimination of two Al–H hydrogens followed by oxidative addition of a C–H bond. (Fig. 7). Reductive elimination from a Ti(iii) system would be unexpected; however, we chose to consider this pathway because of its presence in the literature and because of recent examples of reductive elimination from Al(iii) systems.^10^–^12^ We hypothesized that a σ-bond metathesis mechanism in which one Al–H hydrogen and one C–H hydrogen are eliminated to directly form H_2_ and a C–Al bond might be more probable.

To differentiate between these two mechanisms we synthesized the deuterium labelled analogue of **2**, in which the metal hydrides are replaced with deuterium atoms (**2D**). Upon heating, a reductive elimination mechanism was expected to result in D_2_ loss and leave two bridging deuterium atoms and two bridging hydrogen atoms in **6**, while σ-bond metathesis was expected to produce HD and leave four bridging deuterium atoms on **6** (Fig. 7).

Deuteration of both the alanate (**1D**) and **2D** was confirmed by IR spectroscopy; the strong Al–H stretches around 1646 cm^–1^ in **1** and the Al–H stretch at 1834 cm^–1^ in **2** were absent, and in both cases new peaks around 1200 cm^–1^ were present (see ESI†).

Heating **2D** in deuterated benzene overnight resulted in clean conversion to **6D**. Deuterium labelling experiments were also carried out with **8** by synthesizing **8D** from Cp*_2_TiCl and **1D**. Electron-impact mass spectrometry (EI-MS) of **6D** and **8D** shows a broad isotopic distribution indicative of scrambling of the bridging deuterium atoms throughout the molecule, including the bridging hydride positions as well as the Cp C–H bonds (see ESI†). Furthermore, subjecting **6D** to 1 atm of H_2_ at 100 °C led to production of HD, though no formation of other products was observed. This combined with EI-MS indicates that the deuterium labels are scrambled into the C–H positions in the Cp rings of **6** in solution. While this scrambling inhibits elucidation of previous mechanistic steps, these studies show that bridging hydrides and Cp C–H hydrogens exchange under the reaction conditions (see ESI†).

To further explore the mechanism of dihydrogen elimination, we carried out DFT calculations using the model system Cp_2_TiH_3_AlMe (**2′**) for **2** at the B3LYP/6-31G+(d,p) level of theory and obtained the reaction scheme shown in Fig. S43.† The energetic barriers for both σ-bond metathesis and reductive elimination of dihydrogen were found to be approximately 50 kcal mol^–1^. The magnitude of these numbers likely results from the simplification of a bimolecular pathway to a unimolecular model. While this simplification hinders definitive assignment of a mechanism, these calculations may be used to frame the mechanistic possibilities (for further discussion of these DFT calculations, see section F of the ESI†).

σ-Bond metathesis was found to be the lower energy pathway to the thermal evolution of dihydrogen from **2** and **3**, as expected. Unexpectedly, the ΔΔ*G*^‡^ for reductive elimination *vs.* σ-bond metathesis was calculated to be only 7.5 kcal mol^–1^. Given this small difference in Δ*G*^‡^, we hypothesized it might be possible to favor reductive elimination under a different set of conditions. With the information gained from these DFT calculations, we set out to find conditions under which reductive elimination might occur.

### Oxidation reactivity

Since oxidation of Ti(iii) to Ti(iv) increases the electron deficiency of the complex, reductive elimination modes of reactivity are expected to be enhanced. Initial attempts to synthesize bimetallics from Ti(iv) starting materials were unsuccessful due to the reducing nature of **1**. Instead, we looked to study the reactivity of **2** with oxidizing agents to determine if reductive elimination was possible for a Ti(iv)/Al(iii) complex.

**Scheme 5:**
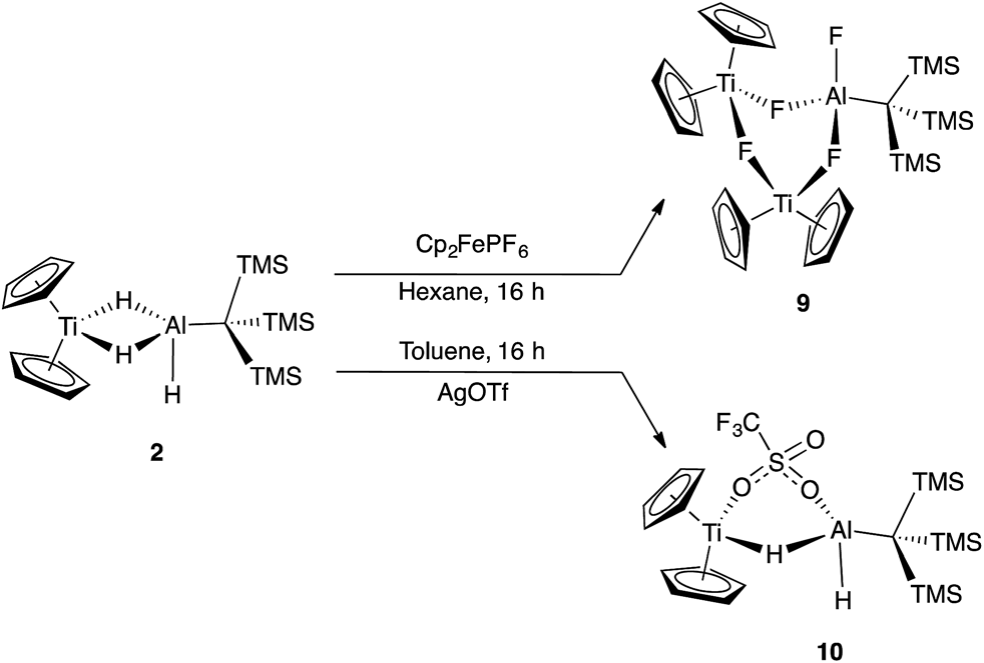
Reactivity of **2** upon treatment with oxidizing agents.

**Fig. 8:**
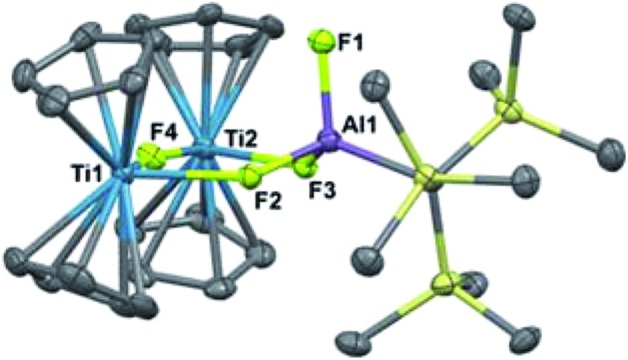
Crystallographically determined structure of **9**. Thermal ellipsoids are shown at the 50% probability level. C–H hydrogen atoms are omitted for clarity. Relevant distances and bond lengths: Ti(1)–Al(1): 3.640(1) Å, Ti(2)–Al(1): 3.643 Å, Ti(1)–Ti(2): 4.085(1) Å, Ti(1)–F(2): 2.156(2) Å, Ti(1)–F(4): 2.051(2) Å, Ti(2)–F(4): 2.060(2) Å, Ti(2)–F(3): 2.186(2) Å, Al(1)–F(1): 1.677(3) Å, Al(1)–F(2): 1.724(2) Å, Al(1)–F(3): 1.725(2) Å.

Reaction of **2** with [Cp_2_Fe][PF_6_] resulted in vigorous bubbling and the formation of a bright green solution. NMR spectroscopy confirmed the formation of dihydrogen and ferrocene. From this reaction, a new species **9** could be crystallized; X-ray structure determination revealed it to be the trimetallic species (Cp_2_Ti)_2_(μ-F)_3_(F)Al(C(TMS)_3_) (Scheme 5 and Fig. 8). Compound **9** is structurally analogous to **3**, with fluorides in place of hydrides. Both titanium centers maintain the formally +3 oxidation state; the total magnetic moment including both titanium centers (2.24(2) *μ*_B_) is higher than that observed for **3** due to the greater the metal–metal separation in **9**, which inhibits antiferromagnetic coupling.

To avoid the fluoride abstraction evident in the formation of **9**, we attempted the reactions of **2** with [Cp_2_Fe][BArF_24_], [Cp_2_Fe][BArF_20_], [Cp_2_Fe][BPh_4_], and CuCl_2_.§
§(BArF_24_ = tetrakis[3,5-bis(trifluoromethyl)phenyl]borate), (BArF_20_ = tetrakis(pentafluorophenyl)borate), (BPh_4_ = tetraphenylborate). In all cases a mixture of products was obtained and no pure materials could be isolated; we note, however, that a resonance corresponding to dihydrogen was observed by ^1^H NMR spectroscopy.

Cyclic voltammetry of **2** in 1,2-difluorobenzene using tetrabutylammonium BArF_20_ as the electrolyte showed an irreversible oxidation at 1.13 V *vs.* Cp_2_Co/Cp_2_Co^+^. The irreversibility of this wave, combined with consistent production of dihydrogen regardless of the oxidizing agent used, suggests that oxidation of Ti(iii) to Ti(iv) prompts dihydrogen evolution to form a highly reactive species capable of activating a variety of counterions.

**Fig. 9:**
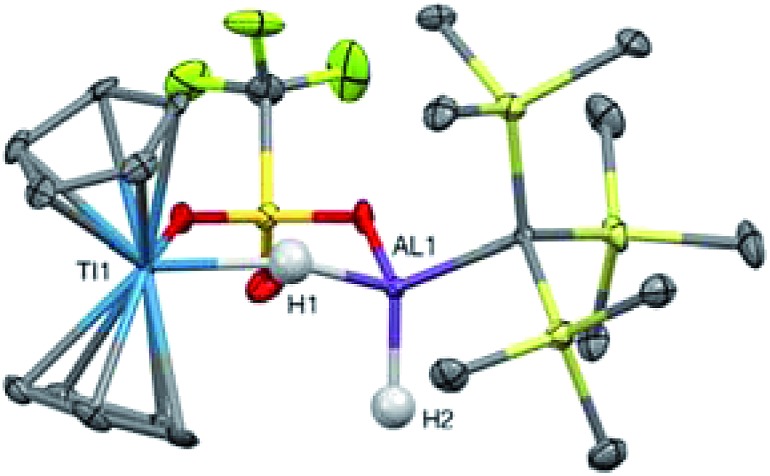
Crystallographically determined structure of **10**. Metal hydrides were located in the Fourier difference map and their positions were refined. C–H hydrogens are omitted for clarity. Thermal ellipsoids are shown at the 50% probability level. Relevant distances and bond lengths: Ti(1)–Al(1): 3.384(1) Å, Ti(1)–O(1): 2.217(2) Å, Al(1)–O(2): 1.876(3) Å.

In an effort to trap a Ti(iv) containing heterometallic, oxidizing agents with coordinating counterions were employed. Reaction of **2** with one equivalent of AgOTf (OTf = trifluoromethanesulfonate) in toluene produced a rapid color change from purple to red, followed by the development of a bright blue color and evolution of gas. A blue product (**10**) was isolated (Scheme 5). Despite a coordinating anion present to stabilize a diamagnetic, oxidized Ti(iv) species, the paramagnetism of the product was evident from ^1^H NMR spectroscopy. The X-ray crystal structure of **10** confirms formation of the Ti(iii) species, Cp_2_Ti(μ-H)(μ-OTf)Al(H)(C(TMS)_3_) in which one of the bridging hydrides in the starting material has been replaced by a triflate (Fig. 9).

**Scheme 6:**
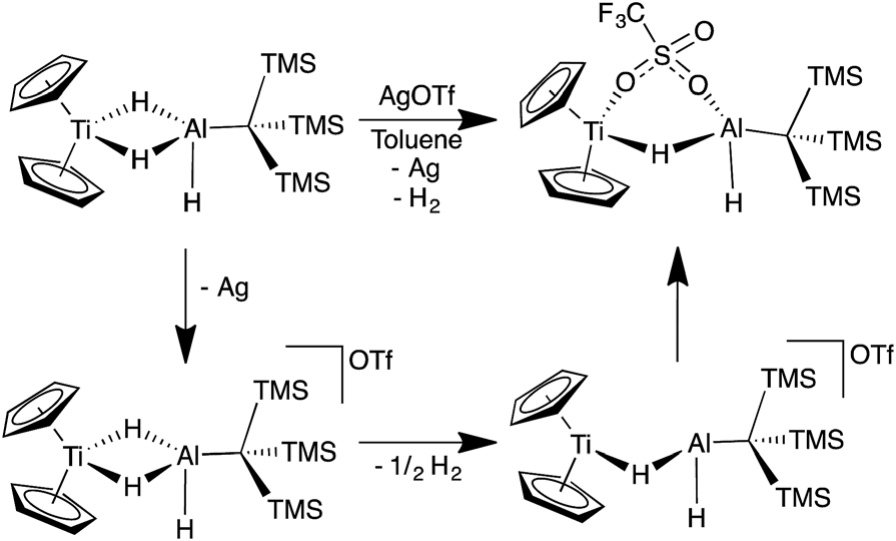
Proposed mechanism for the bimolecular reductive elimination of dihydrogen from **2**. While coordination of triflate subsequent to reductive elimination is depicted, the triflate ion could coordinate before reductive elimination. This difference is likely negligible in nonpolar solvents in which ions are tightly paired.

The formation of **10** and dihydrogen can be rationalized as the reductive elimination of dihydrogen after oxidation by silver. Using data from ^1^H NMR spectroscopy (which showed that H_2_ gas was produced even in deuterated solvents) and EI-MS (which revealed that no deuterium was incorporated into the product; see ESI†), we determined that both the eliminated hydrogen atoms and the remaining hydrides in **10** came from **2** rather than from the solvent. Therefore, we propose that a bimolecular reductive elimination process^65^,^66^ occurs after oxidation of Ti(iii) to Ti(iv), in which two molecules of **2** each eliminate one hydrogen atom. In this process, a one-electron reduction of each heterobimetallic occurs and the complex binds a triflate anion to form the product, **10** (Scheme 6). A related reductive elimination pathway may be operative when non-coordinating anions are used; in these cases, elimination of dihydrogen is followed by activation of the counterion by the coordinatively unsaturated bimetallic, as seen in the formation of **9** (Fig. 8).

**Fig. 10:**
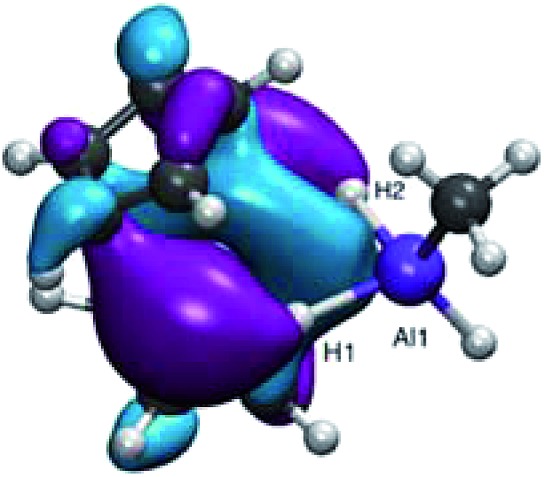
DFT calculated SOMO of **2′**.

From our DFT calculations (see above), the SOMO for **2′** is a Ti-based orbital with Ti–H bonding character (Fig. 10). Removing an electron from this orbital upon oxidation is likely to weaken the Ti–H bonds, facilitating reductive elimination of dihydrogen. This is consistent with the expected behavior for a reductive elimination process; a decrease in electron density at the metal center makes reductive elimination pathways more favorable. While we found that Ti(iii) containing heterometallics may access σ-bond metathesis pathways to activate hydrides at elevated temperatures, reductive elimination was strongly favored upon oxidation to Ti(iv), allowing for dihydrogen formation without ligand activation at room temperature. In contrast to other proposed mechanisms of dihydrogen elimination,^42^ this bimolecular reductive elimination does not require a two-electron reduction at either the transition metal or the aluminum, and therefore provides new opportunities for dihydrogen elimination from systems in which two-electron reductions are difficult to access.

Such redox chemistry bears relevance to the reasons titanium dopants improve H_2_ elimination performance of aluminum hydrogen storage materials. In our molecular systems, oxidation leads to reductive elimination of hydrogen and the formation of a coordinatively unsaturated species that can be trapped with a coordinating counterion. The transfer of electrons away from the titanium–aluminum center may encourage the evolution of hydrogen in materials as well; however, the additional electron density may be delocalized across the material to temper the reactivity of the species following reductive elimination. In general, the reductive elimination of dihydrogen from this titanium–aluminum bimetallic suggests that new chemistry may be accessible from aluminum hydride based molecules and materials, but further investigations are necessary to elucidate the underlying causes of this reactivity. Spectroscopic evaluation of the electronic structure in such bimetallic species will be the focus of future work.

## Conclusions

Stable, yet reactive paramagnetic titanium aluminum heterometallics, including a titanium–aluminum bimetallic complex with bridging hydrides, were synthesized using an alanate supported by the sterically encumbering tris(TMS)methyl ligand. Treatment of the bimetallic complex with diisopropylcarbodiimide resulted in a hydride insertion product while treatment of the bimetallic complex with *tert*-butylisocyanate resulted in transfer of an oxo moiety and isocyanate coupling. Upon heating the heterometallics, C–H bonds in the Cp ligands on titanium were activated; the thermal evolution of dihydrogen likely occurs *via* σ-bond metathesis. Oxidation of the heterobimetallic consistently resulted in the formation of dihydrogen regardless of the oxidizing agent used. Trapping experiments showed that oxidation triggered bimolecular reductive elimination of dihydrogen. Such reactivity may provide insights into the mechanism of hydrogen evolution in hydrogen storage materials.

## Supplementary Material

Supplementary information

Crystal structure data
