# Supplementary material for: Hydride oxidation from a titanium–aluminum bimetallic complex: insertion, thermal and electrochemical reactivity
Source: Chem Sci. 2017 May 31;8(7):5153–60. doi: 10.1039/c7sc01835e (PMC6102661; doi:10.1039/c7sc01835e)
Supplement: Supplementary file 1 [file SC-008-C7SC01835E-s001.pdf]

Electronic Supplementary Material (ESI) for Chemical Science.
This journal is © The Royal Society of Chemistry 2017

Hydride oxidation from a titanium-aluminum bimetallic: insertion, thermal and electrochemical reactivity

Alexandra C. Brown[1], *Alison B. Altman*[1,2], *Trevor D. Lohrey*[1,2], *Stephan Hohloch*[1,2], *John Arnold*[1,2]

[1] Department of Chemistry, University of California, Berkeley, California, 94720; [2]Chemical Sciences Division, Lawrence Berkeley National Laboratory, Berkeley, California 94720

Supporting Information

A. Experimental Details S2-S7
B. NMR Spectra S8-S20
C. Infrared Spectra S21-S27
D. UV-Vis Spectra S28-S29
E. Cyclic Voltammetry S30-S31
F. Computational Details S32-S40
G. Crystallographic Details S41-S43
H. EI Mass Spectrometry Data S44-S46
I. References S47-S48

A. Experimental Details:

General Considerations: Unless otherwise noted, all reactions were performed using standard Schlenk line techniques or in an MBraun inert atmosphere glove box under an atmosphere of nitrogen (<1ppm O_2/H_2O). Glassware and Celite® were stored in an oven at ca. 140 °C. Molecular sieves (4 Å) were activated by heating to 300 °C overnight under vacuum prior to storage in a glovebox. Diethyl ether (Et_2O), tetrahydrofuran (THF), n-hexane and toluene were degassed and dried using a Phoenix solvent drying system commercially available from JC Meyer Solvent Systems and were stored under an atmosphere of nitrogen. Hexamethyldisiloxane (HMDSO) was distilled from sodium/benzophenone, degassed by sparging with nitrogen, and stored over 4 Å molecular sieves. C_6D_6 was dried over Na/benzophenone and degassed by three freeze–pump–thaw cycles before being vacuum transferred and subsequently stored in the glovebox over 4 Å activated molecular sieves. Cp^*_2TiCl[1], Cp_2TiCl[2,3], $(TMS)_3CH$[4], $LiH_3AlC(TMS)_3{\cdot}(THF)_2$[5] Cp_2FePF_6[6] were prepared according to literature procedures. $Cp_2FeBArF_{24}$, $Cp_2FeBArF_{20}$ and Cp_2FeBPh_4 were prepared by the same procedure as for Cp_2FePF_6 from $NaBArF_{24}$[7], $KBArF_{20}$ and $NaBPh_4$, respectively. All other reagents were purchased and used as received with the exception of $LiAlH_4$ which was purified by extraction into Et_2O and potassium bis(trimethylsilyl)amide (KHMDS) which was recrystallized from toluene. Isocyanates and carbodiimides were degassed by three freeze-pump-thaw cycles and stored over sieves. NMR spectra were recorded on Bruker AV-600, AV-500, AVB-400, AVQ-400, AV-300, and DRX-500 spectrometers. ^1H and $^{13}C\{^1H\}$ chemical shifts are given relative to residual solvent peaks. ^{27}Al NMR chemical shifts are referenced to an external standard of 1 M $Al(NO_3)_3$ in H_2O/D_2O (δ 0 ppm). For paramagnetic samples, ^1H NMR integrations were not found to be reliable, and the majority of resonances were difficult to assign. However, the presence of a broad resonance between 0.3 and 0.5 ppm, assigned to the Si-CH_3 protons, was found to be diagnostic of one paramagnetic aluminum species in solution. FT-IR samples were prepared as Nujol mulls and were taken between KBr disks using a Nicolet iS10 FT-IR spectrometer. Diagnostic IR stretches are reported in the experimental details and full IR spectra are provided in the section C. Melting points were determined using an OptiMelt automated melting point system. Elemental analyses were determined at the School of Human Sciences, Science Center, London Metropolitan University. For several samples, satisfactory elemental analyses could not be obtained after multiple attempts, presumably due to minor decomposition from air exposure or slow solvent loss from the crystal lattice during

analysis. For an estimation of the purity of these compounds, see the NMR data reported in section B. X-ray structural determinations were performed at CHEXRAY, University of California, Berkeley on a APEX II QUAZAR or SMART APEX Diffractometer or at the Advanced Light Source (ALS) station 11.3.1 using a silicon-monochromated beam of 16 keV (0.7749 Å) synchrotron radiation. In both cases, diffraction data was collected, integrated, and corrected for absorption using Bruker APEX3 software and its associated modules (SAINT, SADABS). Structural solutions and refinements (on F^2) were carried out using SHELXT and SHELXL-2014 in WinGX. Ellipsoid plots and figures were made using Mercury.

$KH_3AlC(TMS)_3$ (1): Synthesized according to a modification of the $LiH_3AlC(TMS)_3·(THF)_2$ procedure.[5] $(TMS)_3CH$ (7.25 mL, 0.026 mol) was dissolved in 30 mL of THF and methyllithium (1.6 M in Et_2O, 17 mL, 0.027 mol) was added via syringe. The solution was heated to reflux for 16 h after which it was cooled to room temperature and added to $LiAlH_4$ (1.2 g, 0.032 mol) in 5 mL of THF. The solution immediately became cloudy white. This mixture was heated to 60 °C for 2 h after which the solvent was removed *in vacuo*. The solid was triturated twice with 5 mL of toluene before extraction into 100 mL of toluene. This solution was added to KHMDS (5.16 g, 0.026 mol) in 10 mL of toluene and stirred overnight. The solvent was removed *in vacuo* and the solid triturated thoroughly with 5 mL of toluene before it was redissolved in 100 mL of toluene. The cloudy suspension was filtered through Celite® to give a colorless solution. The solvent was removed *in vacuo* and the resulting solid was suspended in hexane and collected on a Schlenk frit. Yield: 5.55 g (71%). ^1H NMR (400 MHz, C_6D_6, 293 K) δ 0.49 ppm (s, 27H, Si-CH_3) ^{27}Al NMR (104 MHz, C_6D_6, 293 K) δ 115.8. FT-IR (KBr, Nujol, cm^{-1}): 1646 (s, br, Al-H stretch) MP: dec. 230-286 °C

$KD_3AlC(TMS)_3$ (1D): Synthesized by the procedure reported for **1**. $(TMS)_3CH$ (3.6 mL, 0.013 mol) was dissolved in 30 mL of THF. Methyllithium (1.6 M in Et_2O, 8.5 mL, 0.014 mol) was added via syringe. The solution was heated to reflux for 16 h. The solution was cooled to room temperature and added to $LiAlD_4$ (0.67 g, 0.016 mol) in 5 mL of THF. The solution immediately turned cloudy white. This mixture was heated to 60 °C for 2 h and the solvent was removed *in vacuo*. The solid was triturated twice with 5 mL of toluene before extraction into 75 mL of toluene. This solution was added to KHMDS (2.59 g, 0.013 mol) in 10 mL of toluene and stirred overnight. The solvent was removed *in vacuo* and the solid triturated thoroughly with 5 mL of toluene before it was redissolved in 75 mL of toluene. The white solution was filtered through Celite® to give a

colorless solution. The solvent was removed *in vacuo* and the resulting solid was suspended in hexane and collected on a Schlenk frit. Yield: 2.7 g (69%). Characterization is identical to that reported for **1** but the IR stretch at 1646 cm^{-1} was absent. (See Figure S30)

$Cp_2TiH_3AlC(TMS)_3$ (2): Cp_2TiCl (0.485 g, 2.27 mmol) and $KH_3AlC(TMS)_3$ (1.41 g, 4.69 mmol) were dissolved in 20 mL of Et_2O cooled to -78 °C. The solution immediately turned bright purple. The mixture was allowed to warm to room temperature and stirred for 30 min. Solvent was removed *in vacuo* and the solid was triturated with 5 mL of hexane. The solid was then suspended in 50 mL of hexane and stirred for 3 h, over which time the solution became blue-purple. The mixture was concentrated to 20 mL and filtered through Celite®. The solvent was removed *in vacuo* to yield blue-purple crystals. Yield: 826 mg (83%). $Cp_2TiH_3AlC(TMS)_3$ obtained contained approximately 5% $KH_3AlC(TMS)_3$ which was not found to impede further reactivity studies. X-ray quality and analytically pure crystals were grown by storage of a concentrated hexane solution at -40 °C (Approximate recovery: 40%). ^1H NMR (500 MHz, C_6D_6, 293 K) δ 0.35 (s, Si-CH_3) $^{13}C\{^1H\}$ NMR (126 MHz, C_6D_6, 293 K) δ 7.2 (Si-CH_3) Anal calcd. (%) for $C_{20}H_{40}AlSi_3Ti$: C, 54.64; H, 9.17 Found: C, 54.63; H, 9.22. MP: dec. 116-148 °C. FT-IR (KBr, Nujol, cm^{-1}): 1834 (m, Al-H stretch). UV-Vis: 529 nm (toluene, ε=3000 L/mol·cm) μ_{eff}=1.70 μ_B

$Cp_2TiD_3AlC(TMS)_3$ (2D): Synthesized according to the procedure reported for **2**. Cp_2TiCl (0.25 g, 1.17 mmol) and $KD_3AlC(TMS)_3$ (0.710 g, 2.34 mmol) were dissolved in 20 mL of Et_2O cooled to -78 °C. The solution immediately turned bright purple. The mixture was allowed to warm to room temperature and stirred for 30 min. Solvent was removed *in vacuo* and the solid was triturated with 5 mL of hexane. The solid was then suspended in 30 mL of hexane and stirred for 3 h, over which time the solution became blue-purple. The mixture was concentrated to 10 mL and filtered through Celite®. The solvent was removed *in vacuo* to yield blue-purple crystals. Characterization is identical to that reported for **2** but the IR stretch at 1834 cm^{-1} was absent. (See Figure S31)

$(Cp_2Ti)_2H_4AlC(TMS)_3$ (3): Cp_2TiCl (250 mg, 1.06 mmol) and $KH_3AlC(TMS)_3$ (161 mg, 0.535 mmol) were dissolved in 10 mL of hexane. The purple solution was stirred for 4 h and then filtered through Celite®. The resulting pink-purple solution was concentrated to 5 mL and stored overnight at -40 °C to yield dark purple crystals. Yield: 58 mg (32%). X-ray quality crystals were grown by vapor diffusion of HMDSO into a concentrated hexane solution at room temperature over 3 d. ^1H NMR (500 MHz, C_6D_6, 293 K) δ 0.33 (s, Si-CH_3) $^{13}C\{^1H\}$ NMR (126 MHz, C_6D_6, 293 K) δ 8.1

(Si-CH_3), 100.4 (C-Cp), ^{27}Al NMR (130 MHz, C_6D_6, 293 K) δ 128.5 MP: dec. 177-223 °C. FT-IR (KBr, Nujol, cm^{-1}): 1811 (m, Al-H stretch). μ_{eff}=1.66 μ_B

$Cp_2TiH((^iPr)N(CH)N(^iPr))HAlC(TMS)_3$ (4): $Cp_2TiH_3AlC(TMS)_3$ (195 mg, 0.444 mmol) was dissolved in 5 mL of hexane and a solution of diisopropylcarbodiimide (165 mg, 1.31 mmol) in 2 mL of hexane was added. The mixture was stirred for 1 h before the solvent was removed *in vacuo*. The solids were dissolved in 10 mL pentane and the solution was concentrated to 5 mL and stored at -40 °C overnight to yield blue crystals (50 mg). A second crop of crystals could be obtained through further concentration of the solution (25 mg). Yield: 75 mg (30%). ^{1}H NMR (600 MHz, C_6D_6, 293 K) δ 0.44 (s, Si-CH_3), 1.03, 1.73, 5.09. MP: dec. 132-138 °C. FT-IR (KBr, Nujol, cm-1): 1783 (m), 1666 (m, br), 1595 (m) (N=C and Al-H stretches). Anal calcd. (%) for $C_{27}H_{54}AlN_2Si_3Ti$: C, 57.31; H, 9.62; N, 4.95 Found: C, 57.40; H, 9.69; N, 4.85.

$Cp_2TiOAl(OCHN^tBuC(N^tBuO)(C(TMS)_3)$ (5): $Cp_2TiH_3AlC(TMS)_3$ (200 mg, 0.455 mmol) was dissolved in 5 mL of hexane and a solution of ^{t}BuNCO (135 mg, 1.365 mmol) in 2 mL of hexane was added. The mixture was stirred for 1 h, then the solution was filtered. The solvent was removed *in vacuo* and the solids were dissolved in 10 mL of toluene. The solution was concentrated to 5 mL and stored at -40 °C overnight to yield green crystals. Yield: 72 mg (24%). ^{1}H NMR (600 MHz, C_6D_6, 293 K) δ 0.30 (s, Si-CH_3), 0.79, 2.44 MP: dec. 103-145 °C. FT-IR (KBr, Nujol, cm^{-1}): 1687 (m), 1600 (m), 1566 (m) (O=C and N=C stretches).

$((Cp)(C_5H_4)TiH_2AlC(TMS)_3)_2$ (6): Solid $Cp_2TiH_3AlC(TMS)_3$ (260 mg, 0.591 mmol) was heated to 150 °C for 20 min. During this time the purple solid melted and gas evolution and a color change to dark blue was observed. The solid was cooled to room temperature and extracted with 30 mL hexane, concentrated to 10 mL, and stored at -40 °C overnight to yield dark blue crystals suitable for X-ray analysis. Yield: 86 mg (33%) Higher yields are possible upon further concentration of the solution. ^{1}H NMR (500 MHz, C_6D_6, 293 K) δ 0.43 (s, Si-CH_3) $^{13}C\{^1H\}$ NMR (126 MHz, C_6D_6, 293 K) δ 12.8 (Si-CH_3) MP: dec. 220-246 °C. FT-IR (KBr, Nujol, cm-1): 1640 (m, M-H-M stretch). UV-Vis: 600 nm (toluene, ε=628 L/mol·cm)

$((Cp)(C_5H_4)TiD_2AlC(TMS)_3)_2$ (6D): $Cp_2TiH_3AlC(TMS)_3$ (50 mg, 0.113 mmol) was heated to 100 °C in 0.7 mL of C_6D_6 for 2 h. The solvent was removed *in vacuo*. The crude solid was analyzed using EI-MS (See Figure S42)

$((Cp)(C_5H_4)Ti)_2H_2AlC(TMS)_3$ (7): $(Cp_2Ti)_2H_4AlC(TMS)_3$ (230 mg, 0.372 mmol) was dissolved in 20 mL of toluene and heated to 80 °C for 16 h. Over this time the color changed from purple to

reddish-brown. The solvent was removed *in vacuo* and the solid was extracted into 15 mL of hexane. The solution was concentrated to 10 mL and stored at -40 °C overnight to yield brown crystals. Yield: 123 mg (54%). X-ray quality crystals were grown by vapor diffusion of hexane into a concentrated toluene solution at -40 °C. ^1H NMR (500 MHz, C_6D_6, 293 K) δ 0.16 (s, Si-CH_3) ^{27}Al NMR (130 MHz, C_6D_6, 293 K) δ -134. MP: dec. 215-250 °C. FT-IR (KBr, Nujol, cm^{-1}): 1579 (m, Ti-H-M stretch). UV-Vis: 454 nm (toluene, ε=271 L/mol·cm).

(Cp*)($C_5Me_4CH_2$)TiH_2AlC(TMS)$_3$ (8): Cp*TiCl (501 mg, 1.41 mmol) and LiH_3AlC(TMS)$_3$·(THF)$_2$ (590 mg, 1.43 mmol) were dissolved in 20 mL of Et_2O. The solution was stirred for 10 d at room temperature, over which time the solution turned cloudy and green. The solvent was removed *in vacuo* and the solids were extracted into 20 mL of hexane. The solution was filtered through Celite® and the resulting green solution was concentrated to 15 mL and stored at -40 °C overnight to yield dark green crystals of suitable quality for X-ray analysis. Yield: 395 mg (49%) ^1H NMR (500 MHz, C_6D_6, 293 K) δ 0.38 (s, Si-CH_3), MP: dec. 174-241 °C. Anal calcd. (%) for $C_{30}H_{58}AlSi_3Ti$: C, 62.35; H, 10.12 Found: C, 62.22; H, 9.99.

(Cp*)($C_5Me_4CH_2$)TiD_2AlC(TMS)$_3$ (8D): Cp*TiCl (46 mg, 0.13 mmol) and KD_3AlC(TMS)$_3$ (40 mg, 0.13 mmol) were dissolved in 5 mL of Et_2O. The solution was stirred for 5 d at room temperature, over which time the solution turned cloudy and green. The solvent was removed *in vacuo* and the solids were extracted into 5 mL of hexane. The solution was filtered through Celite® and the resulting green solution was concentrated to 2 mL and stored at -40 °C overnight to yield dark green crystals. The crystals were analyzed using EI-MS (See Figure S43)

(Cp_2Ti)$_2F_4$AlC(TMS)$_3$ (9): Cp_2TiH_3AlC(TMS)$_3$ (195 mg, 0.444 mmol) and Cp_2FePF_6 (147 mg, 0.444 mmol) were suspended in 10 mL of hexane. The solution rapidly became dark green and gas evolution was observed; the mixture was stirred for 16 h. The solution was then filtered to yield a bright green solution. This solution was concentrated to 5 mL and stored at -40 °C overnight to yield green crystals. Yield: 76 mg (50%). X-ray quality crystals were grown by vapor diffusion of HMDSO into a concentrated hexane solution at room temperature. ^1H NMR (500 MHz, C_6D_6, 293 K) δ 0.33 (s, Si-CH_3) $^{13}C\{^1H\}$ NMR (126 MHz, C_6D_6, 293 K) δ 5.51 (Si-CH_3). Anal calcd. (%) for $C_{30}H_{47}AlF_4Si_3Ti_2$: C, 52.17; H, 6.86 Found: C, 51.97; H, 6.92. MP: dec. 218-280 °C μ_{eff}=2.24 μ_B

Cp_2TiH(OTf)AlH(C(TMS)$_3$) (10): In a darkened glovebox, Cp_2TiH_3AlC(TMS)$_3$ (200 mg, 0.455 mmol) was dissolved in 5 mL of toluene and added to AgOTf (117 mg, 0.455 mmol) in 2 mL of

hexane. The mixture was stirred in darkness for 16 h and then the solution was filtered. The solvent was removed *in vacuo* and the resulting solid was dissolved in 10 mL of hexane. The solution was concentrated to 5 mL and stored at -40 °C overnight to yield blue crystals. Yield: 109.4 mg (40%). X-ray quality crystals were grown by slow evaporation of hexane at -40 °C. ^{1}H NMR (500 MHz, C_6D_6, 293 K) δ 0.40 (s, Si-CH_3). FT-IR (KBr, Nujol, cm-^{1}): 1860 (m, H-Al stretch), 1633 (m, S=O stretch). MP: 126-129 °C.

Deuterium labelling of 10: In an NMR tube in a darkened glovebox, $Cp_2TiH_3AlC(TMS)_3$ (26.2 mg, 0.059 mmol) in 0.4 mL of d^8-toluene was added to a solution of AgOTf (15.6 mg, 0.059 mmol) in 0.4 mL of d^8-toluene. The solution was allowed to sit for 16 h in the dark. Over this time, the solution turned blue and silver metal precipitated. A ^{1}H NMR spectrum was recorded (Figure S24). The solution was removed from the NMR tube and the NMR tube was washed with 2 mL of hexane. The solution and washings were combined and filtered through Celite®. The solvent was removed *in vacuo* and the remaining blue solid was analyzed using EI-MS (Figure S44)

B. NMR Spectra

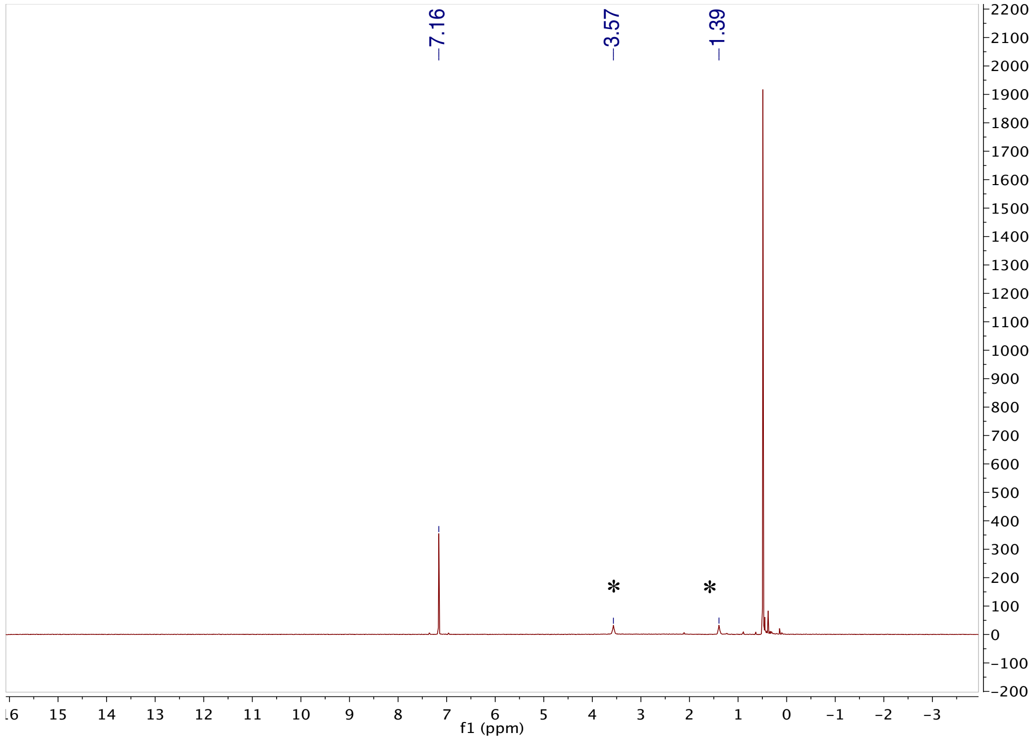

Figure S1: ^{1}H NMR Spectrum of **1** in C_6D_6 at 293 K *residual THF

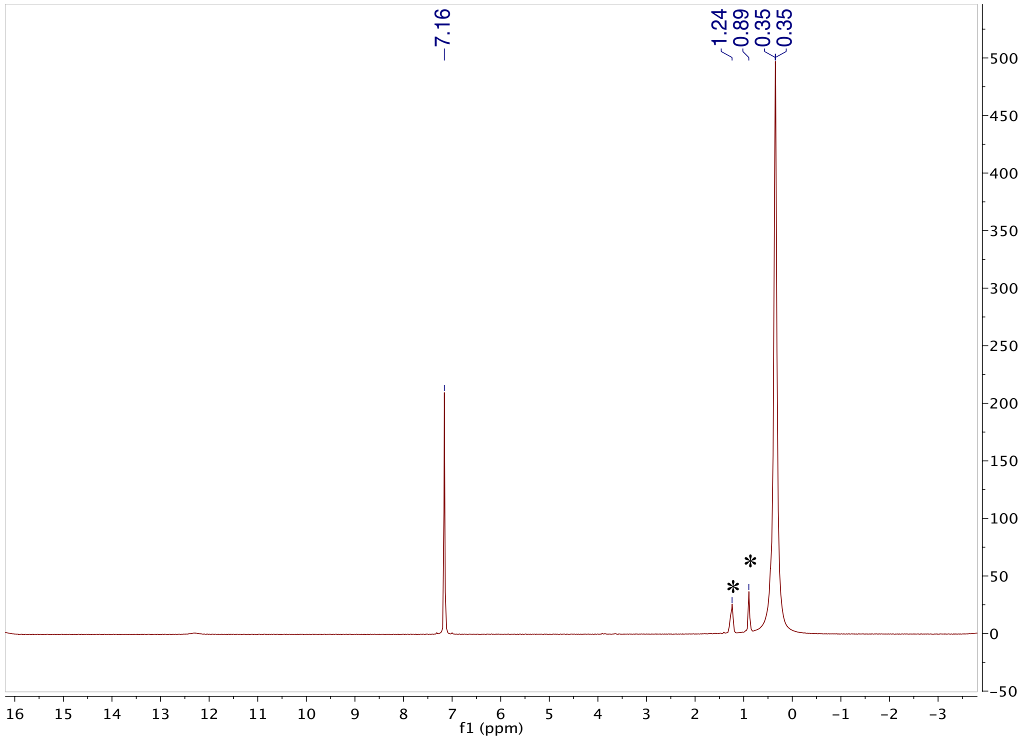

Figure S2: ^{1}H NMR Spectrum of **2** in C_6D_6 at 293 K *residual hexane

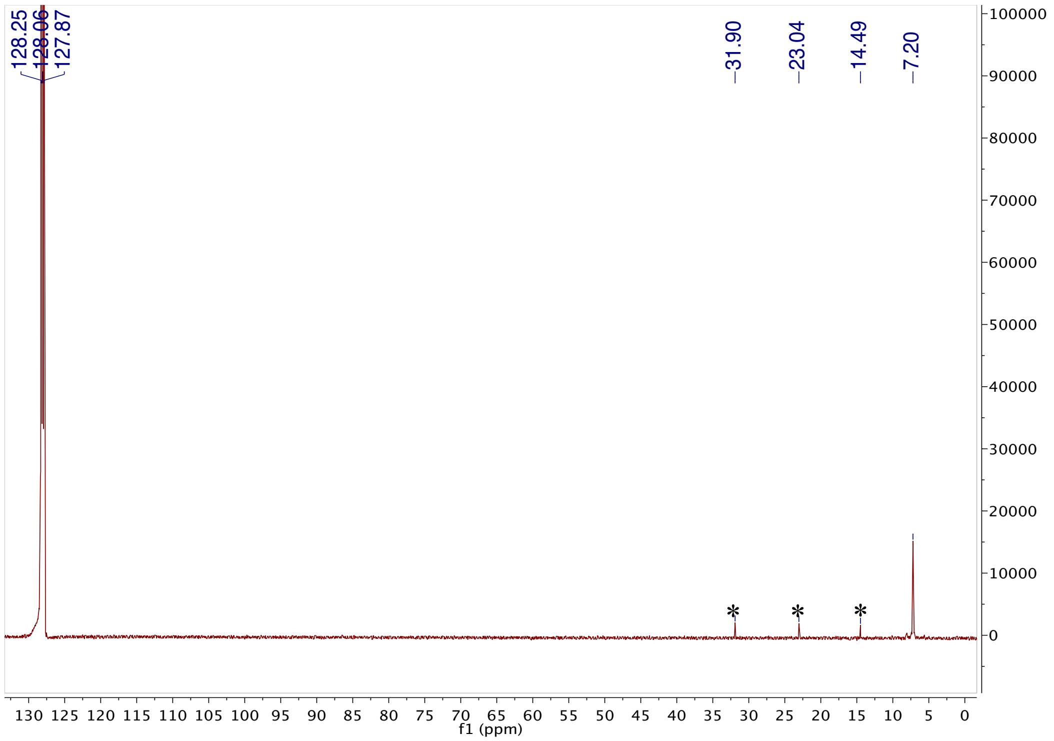

Figure S3: ^{13}C NMR Spectrum of **2** in C_6D_6 at 293 K *residual hexane

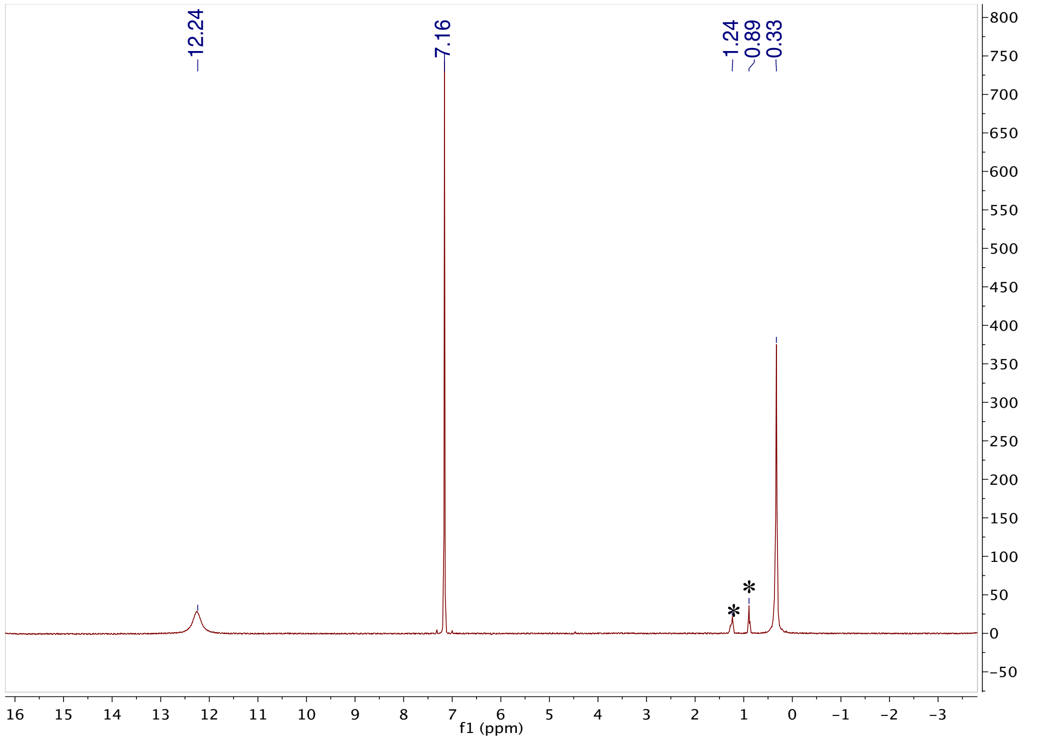

Figure S4: ^{13}C NMR Spectrum of **3** in C_6D_6 at 293 K *residual hexane

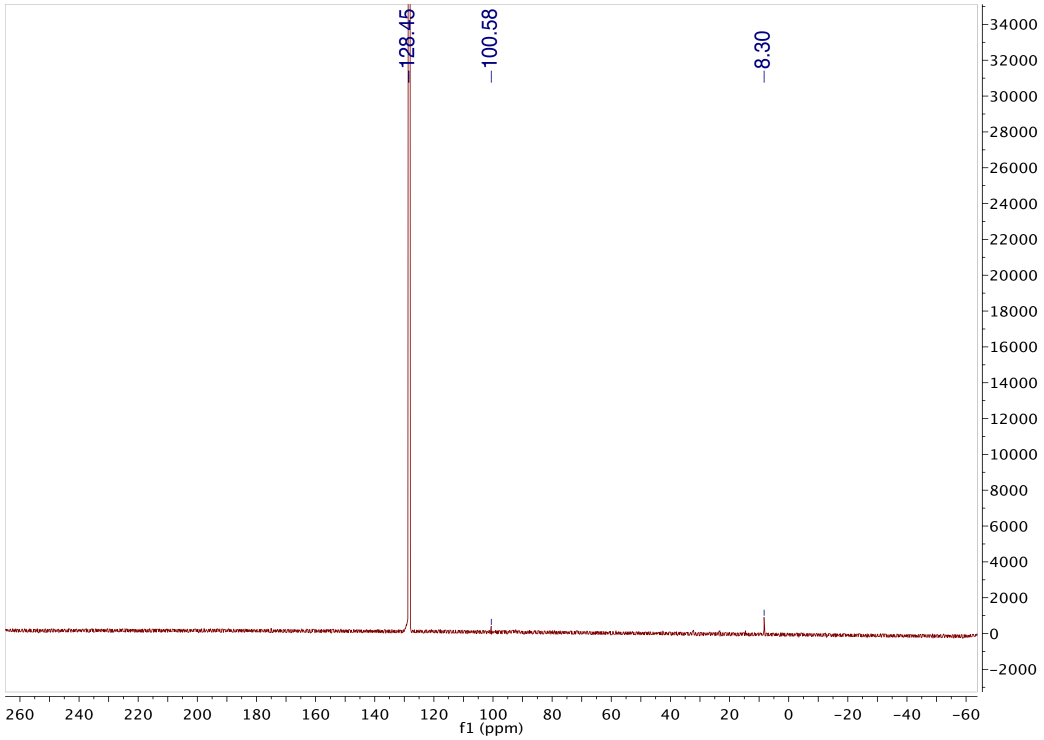

Figure S5: ^{13}C NMR Spectrum of **3** in C_6D_6 at 293 K

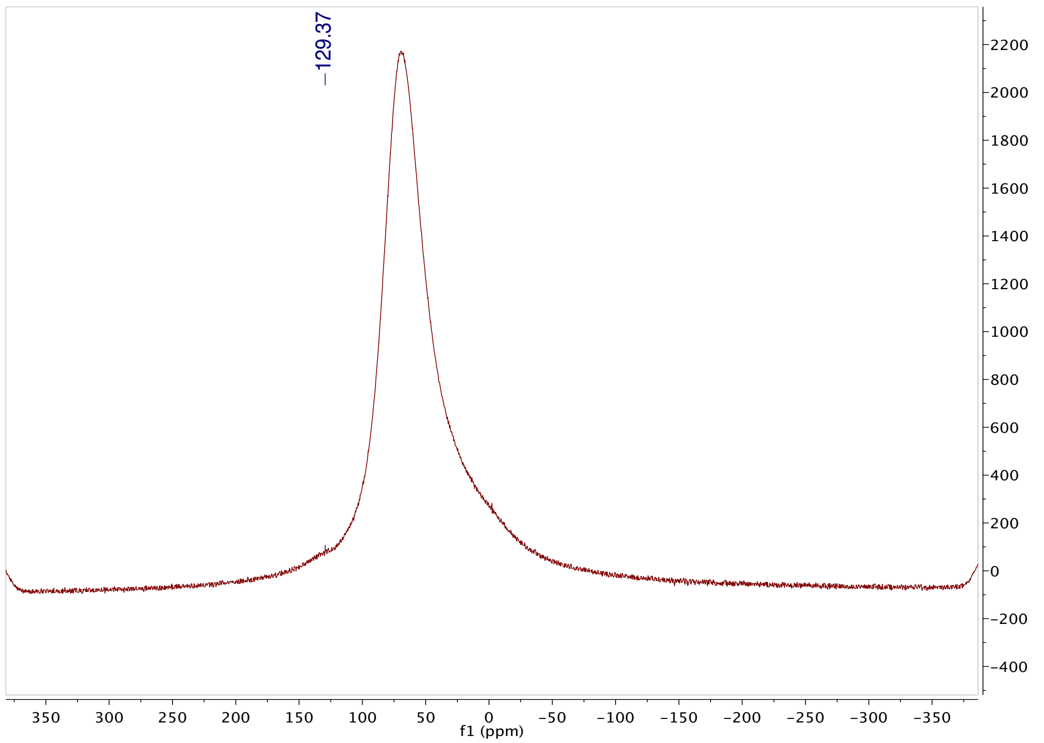

Figure S6: ^{27}Al NMR Spectrum of **3** in C_6D_6 at 293 K

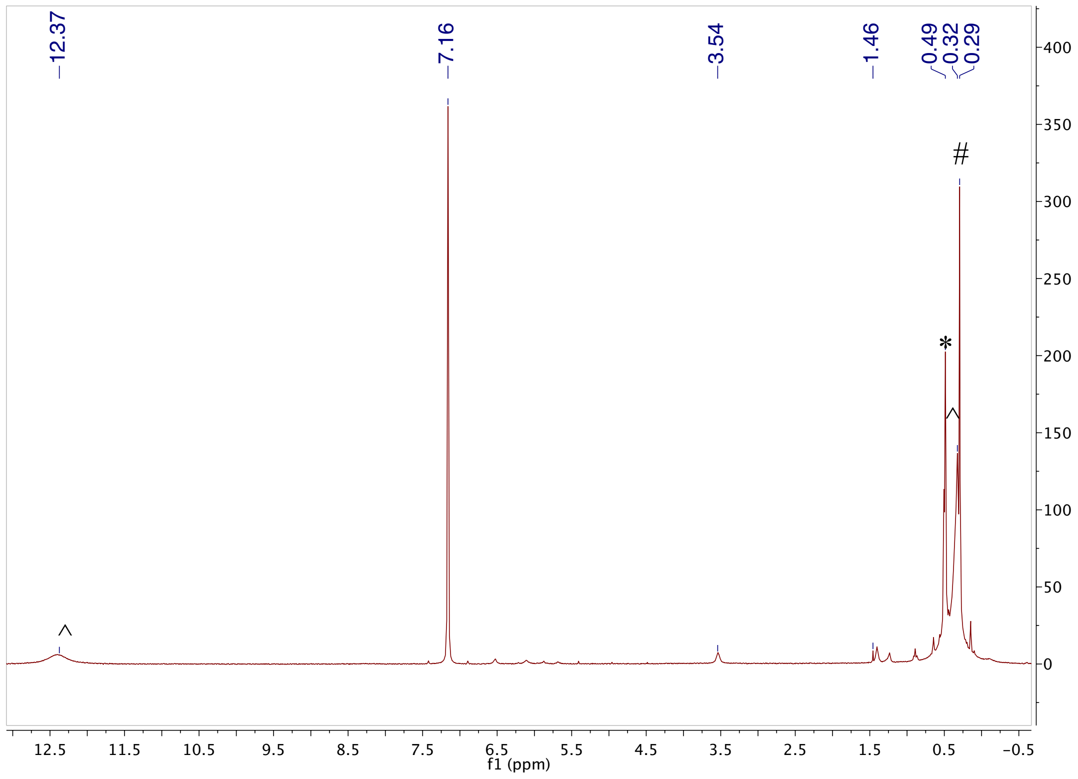

Figure S7: ^{1}H NMR Spectrum of **2** and KH in C_6D_6 at 293 K showing formation of **1** (*) and **3** (^) (# grease)

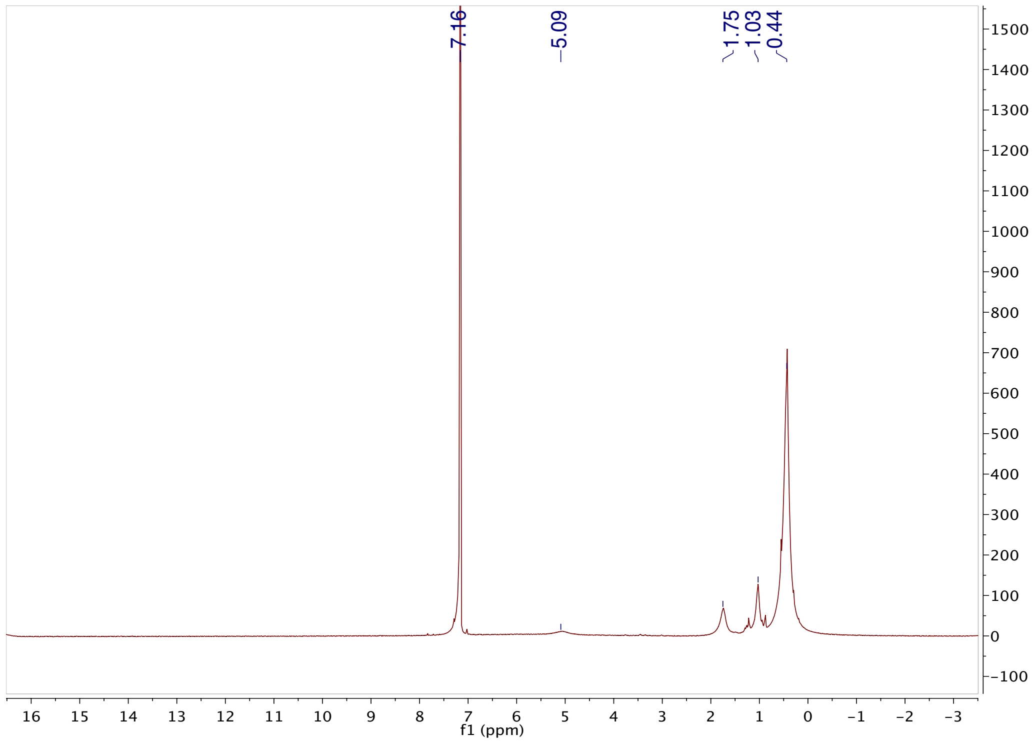

Figure S8: ^{1}H NMR Spectrum of **4** in C_6D_6 at 293 K

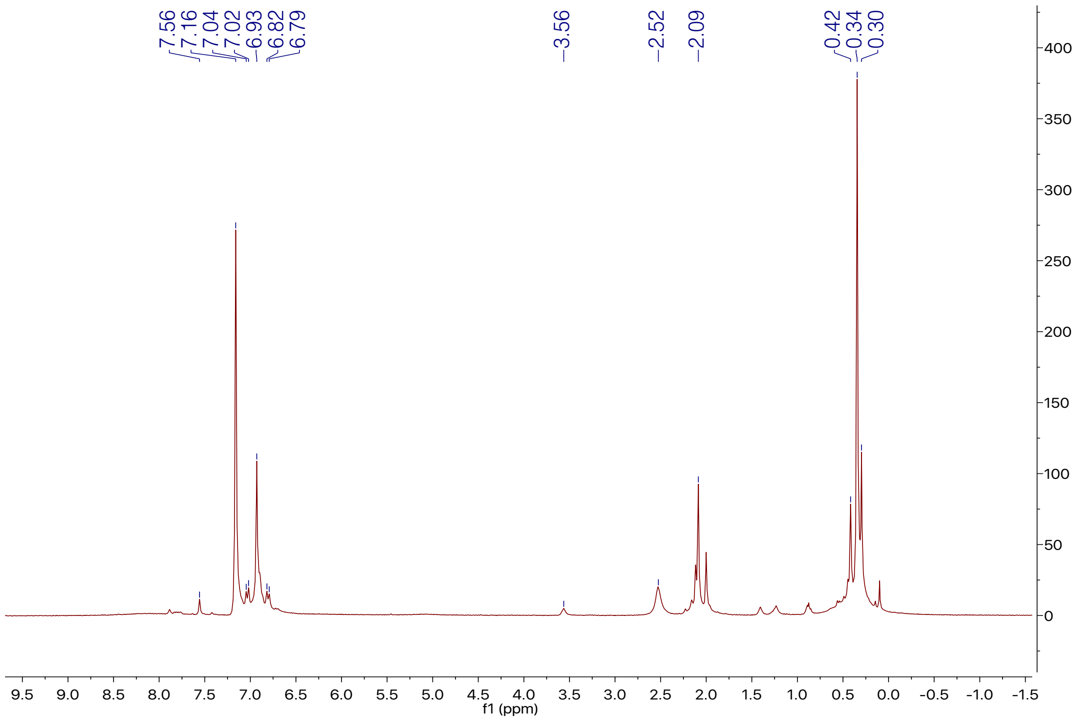

Figure S9: ^{1}H NMR Spectrum of **3** and di(p-tolyl)carbodiimide in C_6D_6 at 293 K. No starting material remains

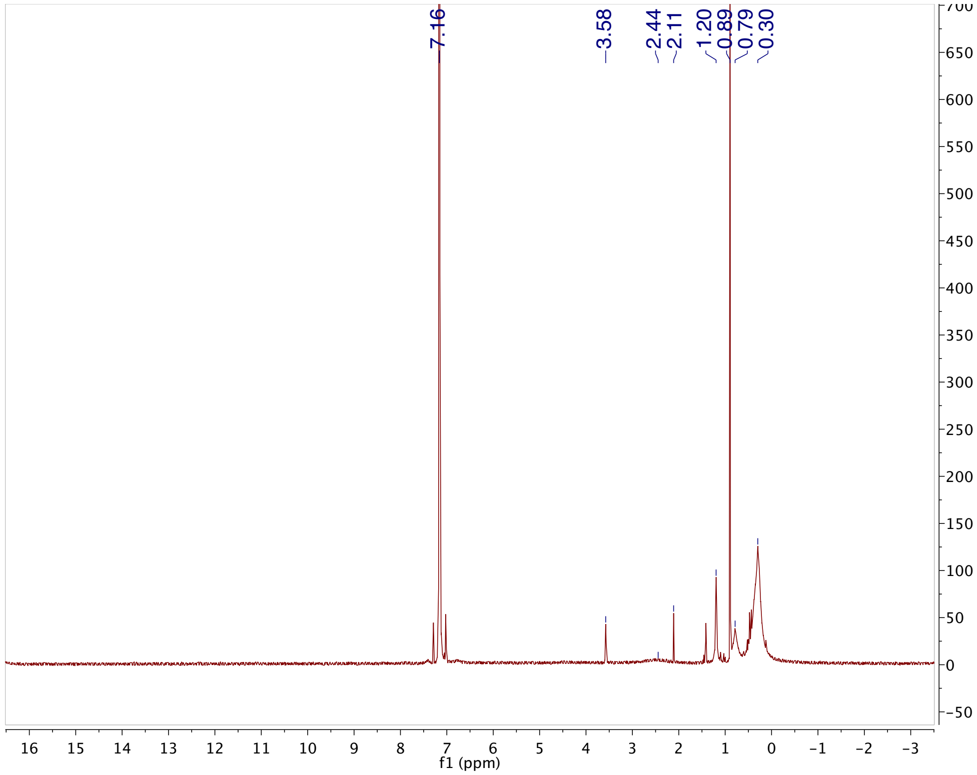

Figure S10: ^{1}H NMR Spectrum of **9** in C_6D_6 at 293 K *residual Et_2O ^residual toluene # ^{t}BuNCO

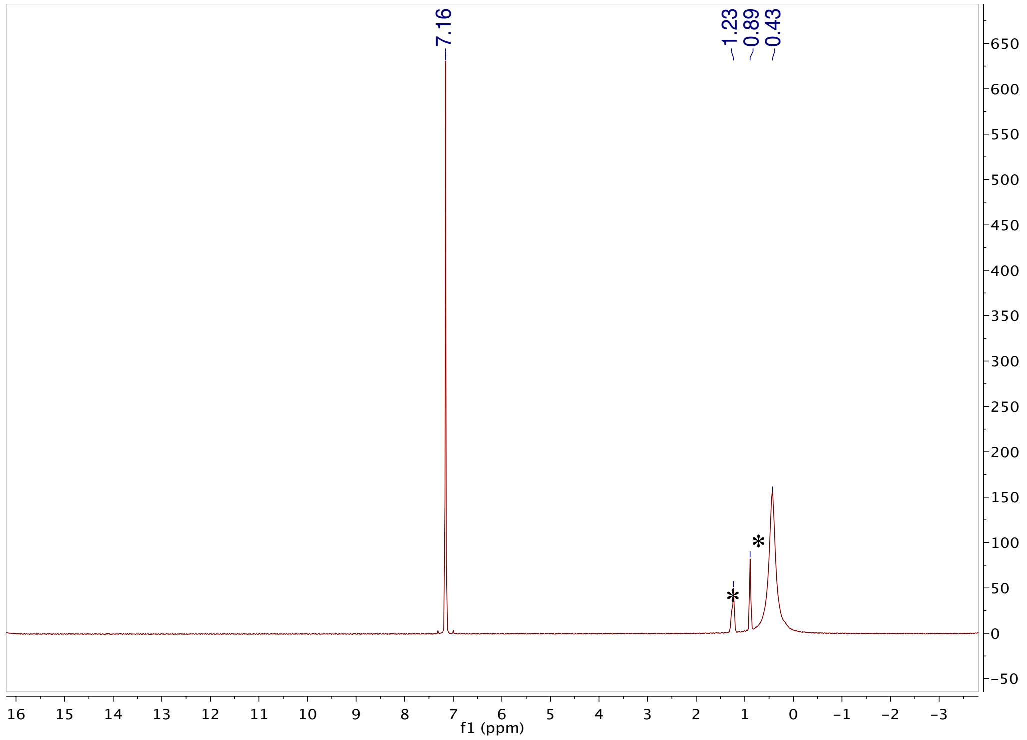

Figure S11: ^1H NMR Spectrum of **6** in C_6D_6 at 293 K *residual hexane

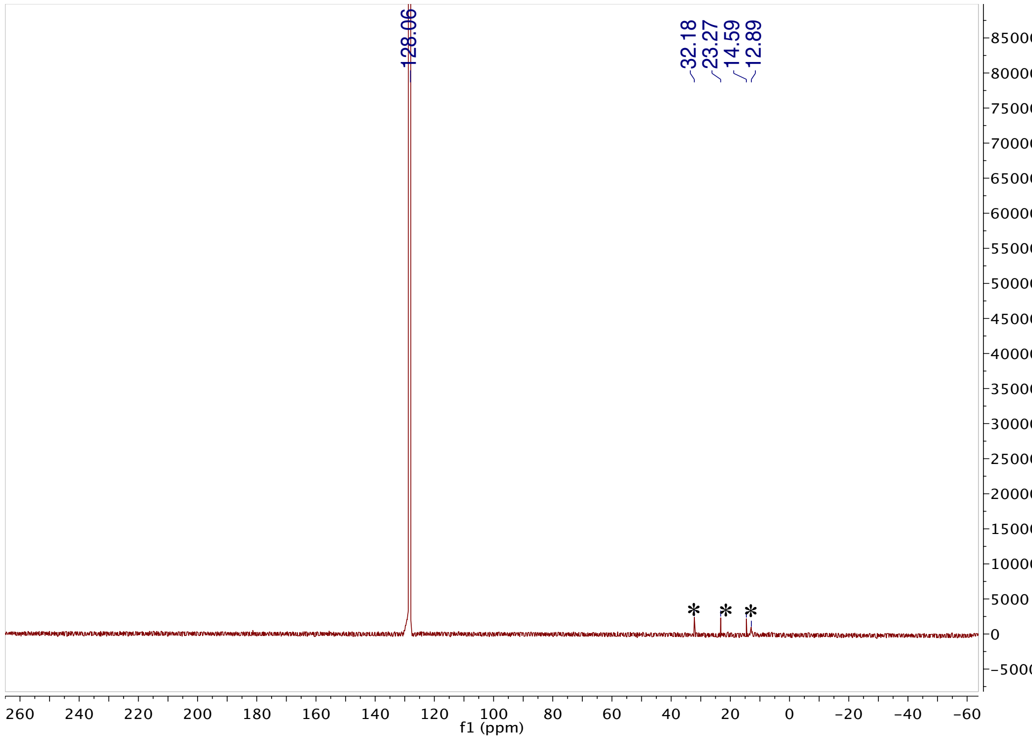

Figure S12: ^{13}C NMR Spectrum of **6** in C_6D_6 at 293 K *residual hexane

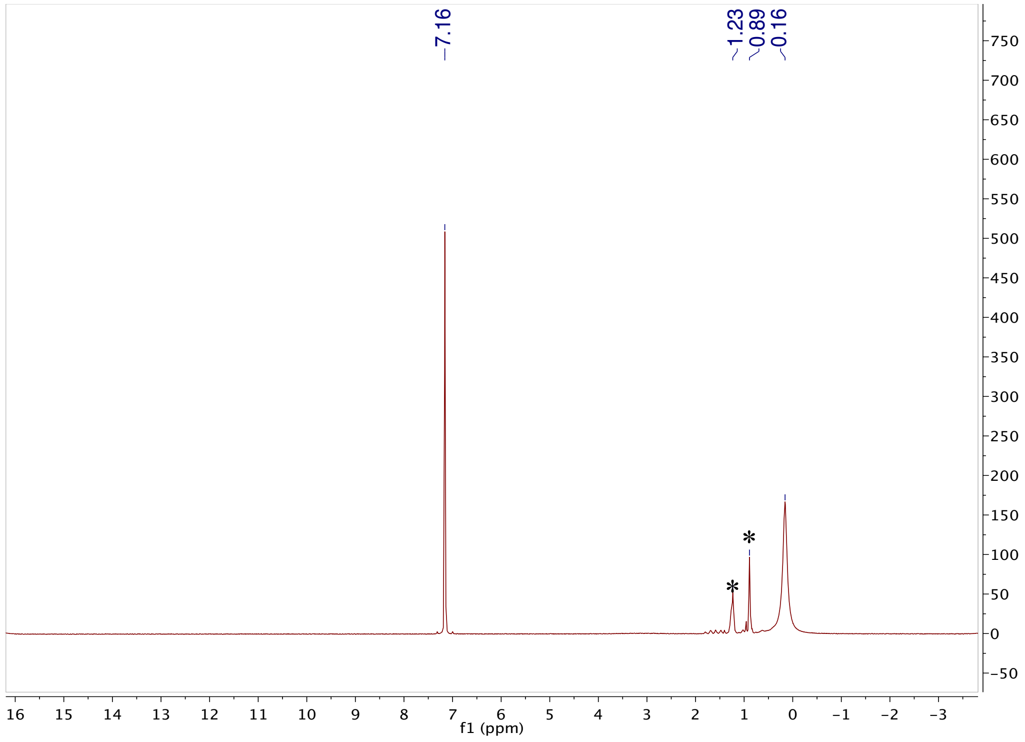

Figure S13: ^{1}H NMR Spectrum of **7** in C_6D_6 at 293 K *residual hexane

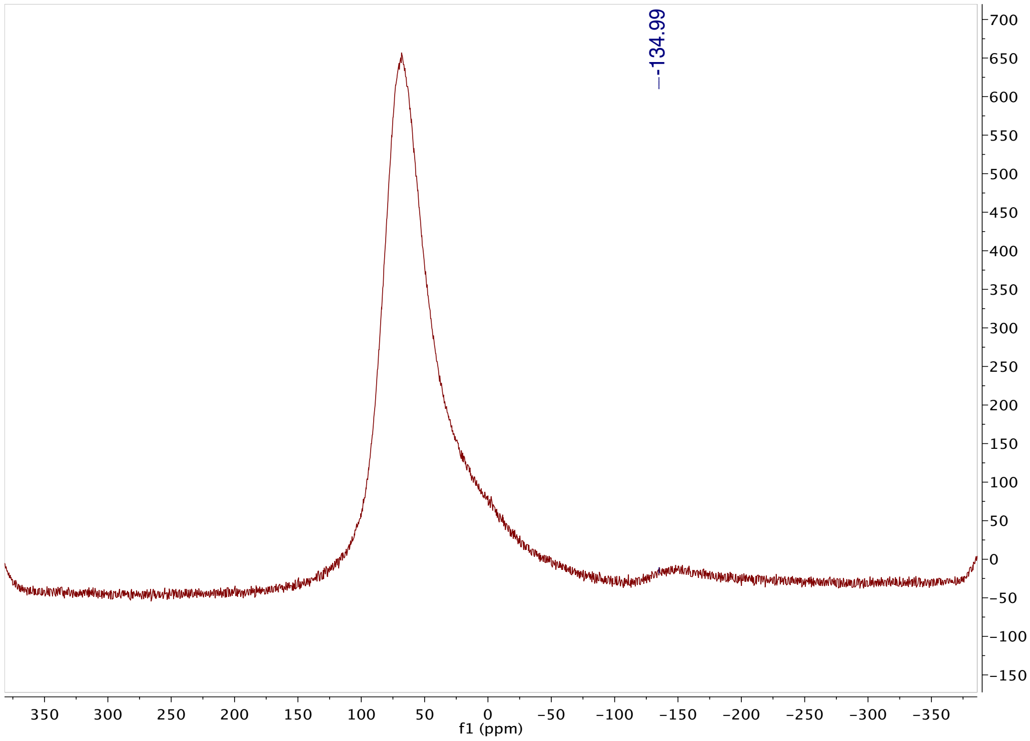

Figure S14: ^{27}Al NMR Spectrum of **7** in C_6D_6 at 293 K

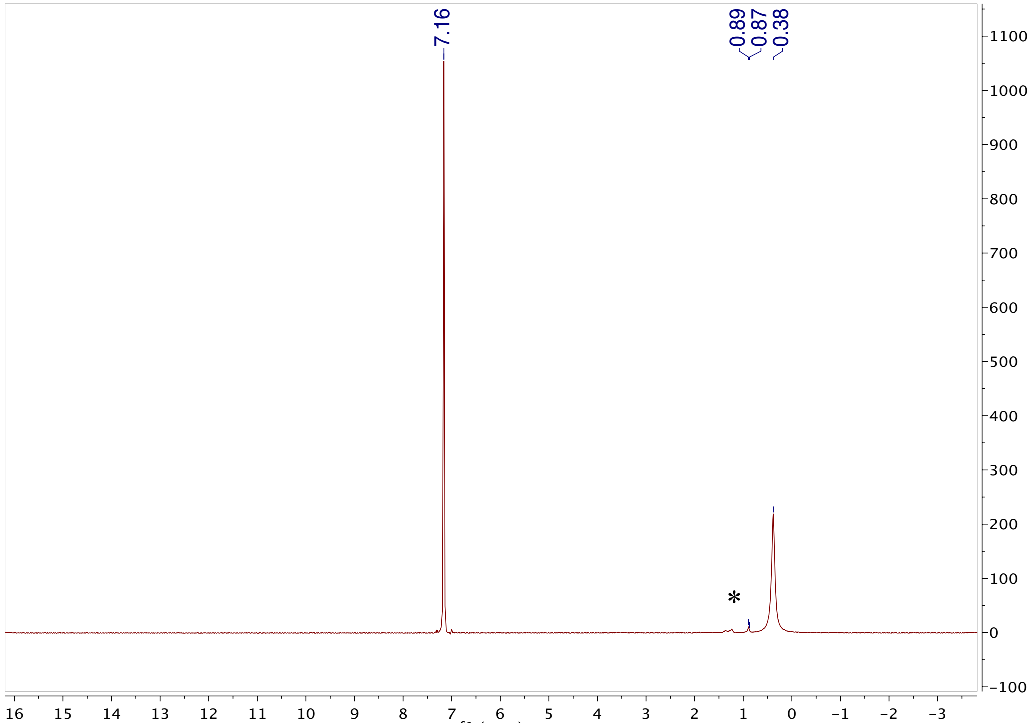

Figure S15: ^{1}H NMR Spectrum of **8** in C_6D_6 at 293 K *residual hexane

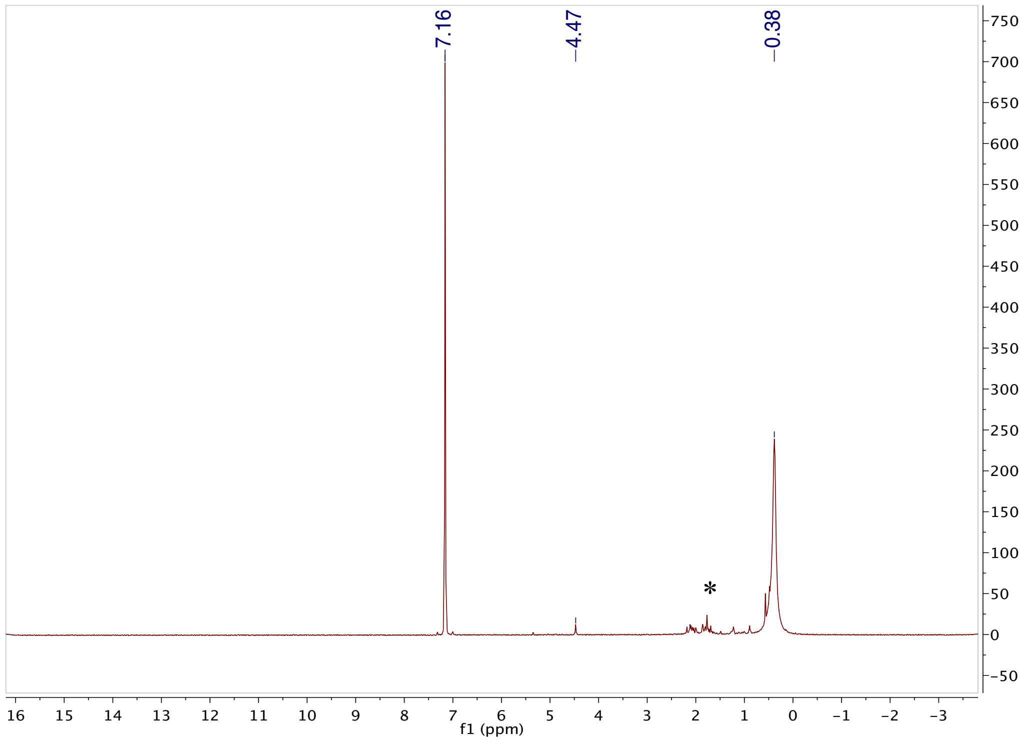

Figure S16: ^{1}H NMR Spectrum of **8** in C_6D_6 at 293 K after addition of H_2 and heating for 16 h. Minor decomposition (*) was observed immediately and did not change during the experiment

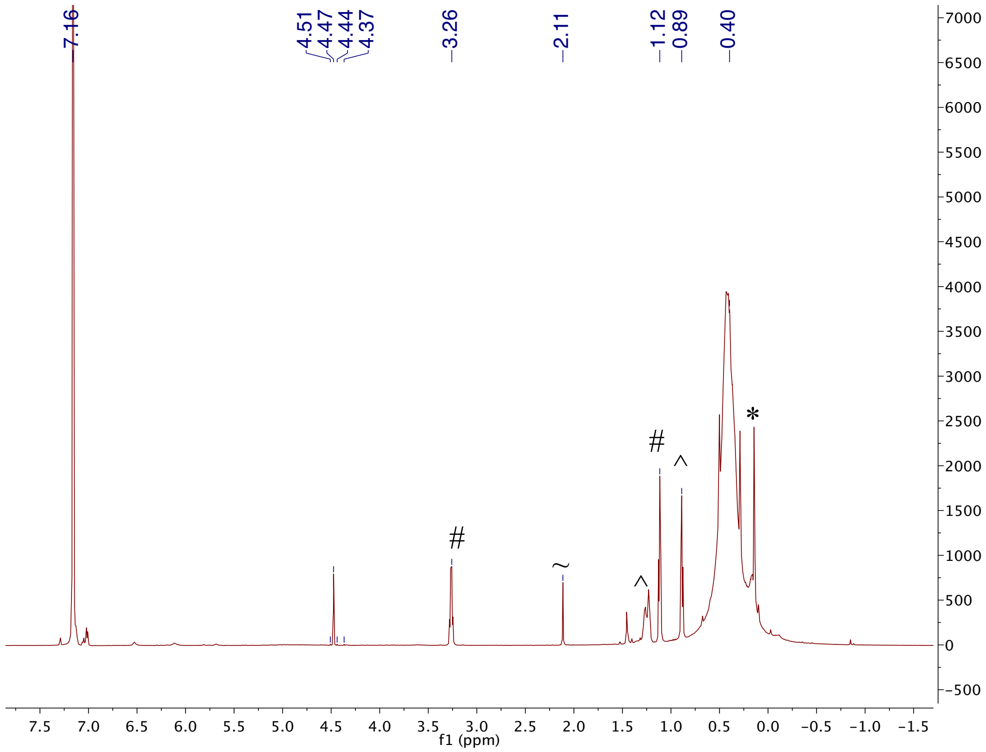

Figure S17: ^{1}H NMR Spectrum of **6D** in C_6D_6 at 293 K after addition of H_2 and heating to 100 °C for 4 h. Minor decomposition (*) was observed immediately and did not change during the experiment (^: residual hexane #: Et_2O, ~: toluene)

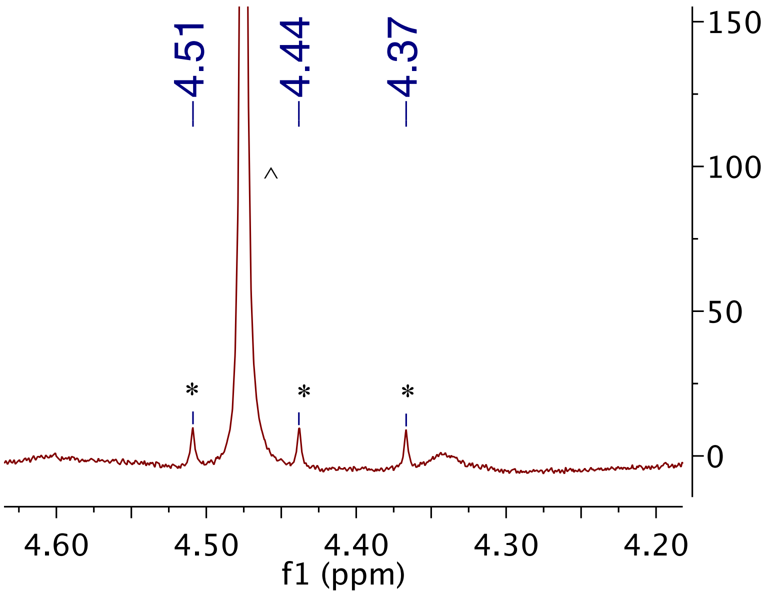

Figure S18: Inset of the ^{1}H NMR Spectrum of **6D** after addition of H_2 and heating to 100 °C for 4 h, showing the presence of H_2 (^) and HD (*)

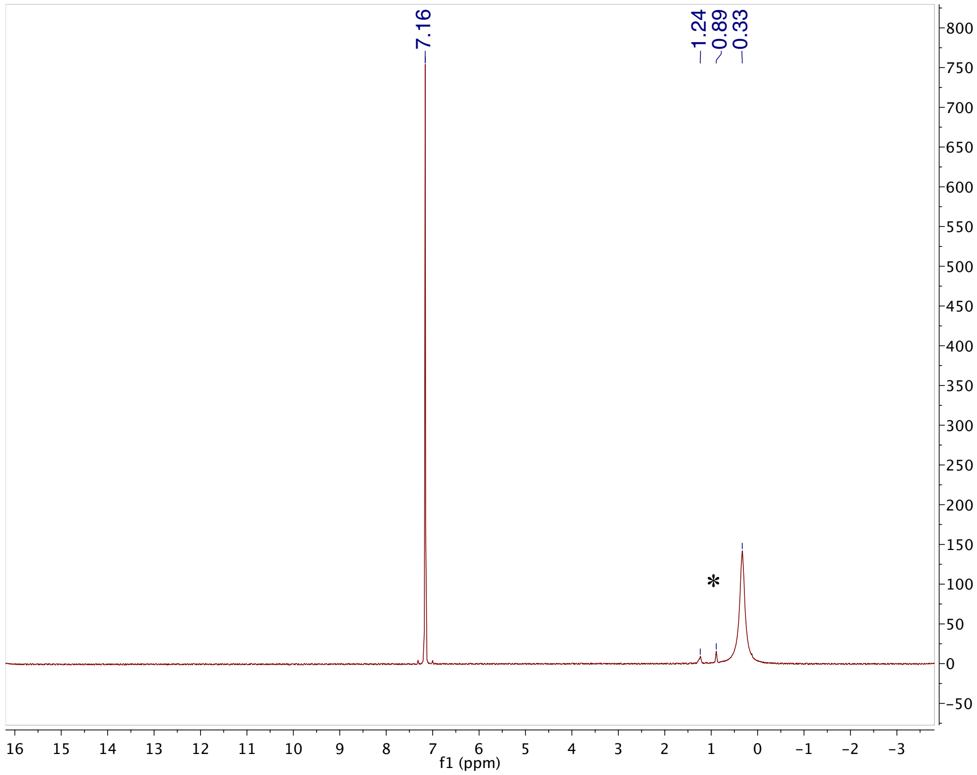

Figure S19: ^{1}H NMR Spectrum of **9** in C_6D_6 at 293 K*residual hexane

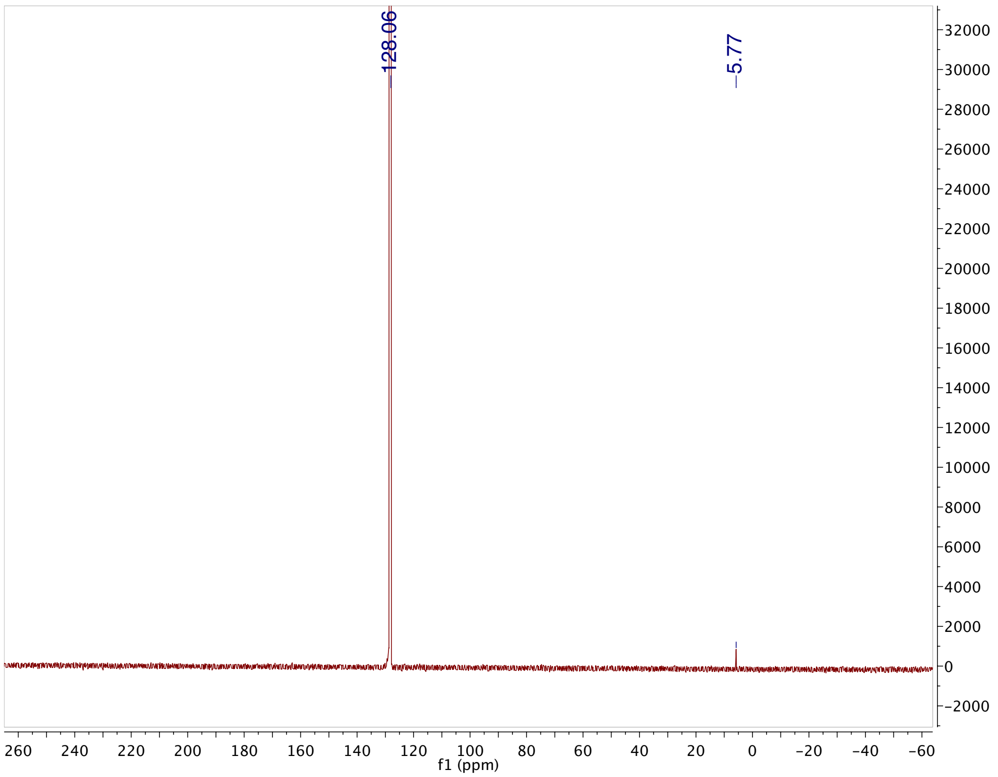

Figure S20: ^{13}C NMR Spectrum of **9** in C_6D_6 at 293 K

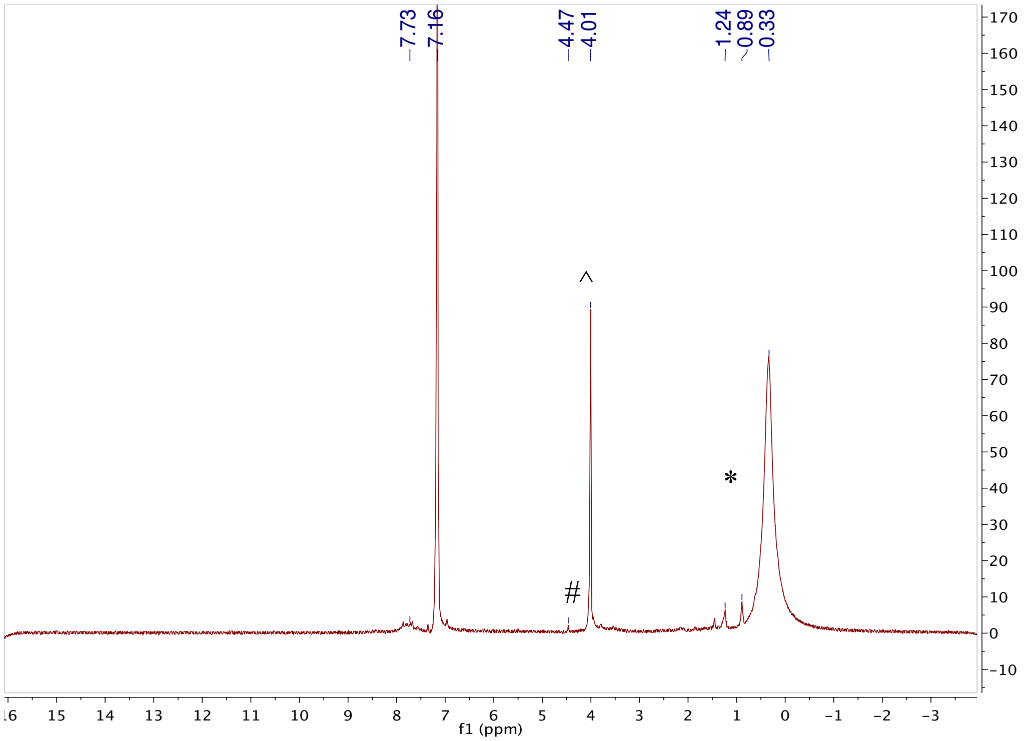

Figure S21: ^{1}H NMR Spectrum of **2** and $Cp_2FeBArF_{24}$ in C_6D_6 at 293 K showing the formation of ferrocene (^) hydrogen (#) and residual hexane (*)

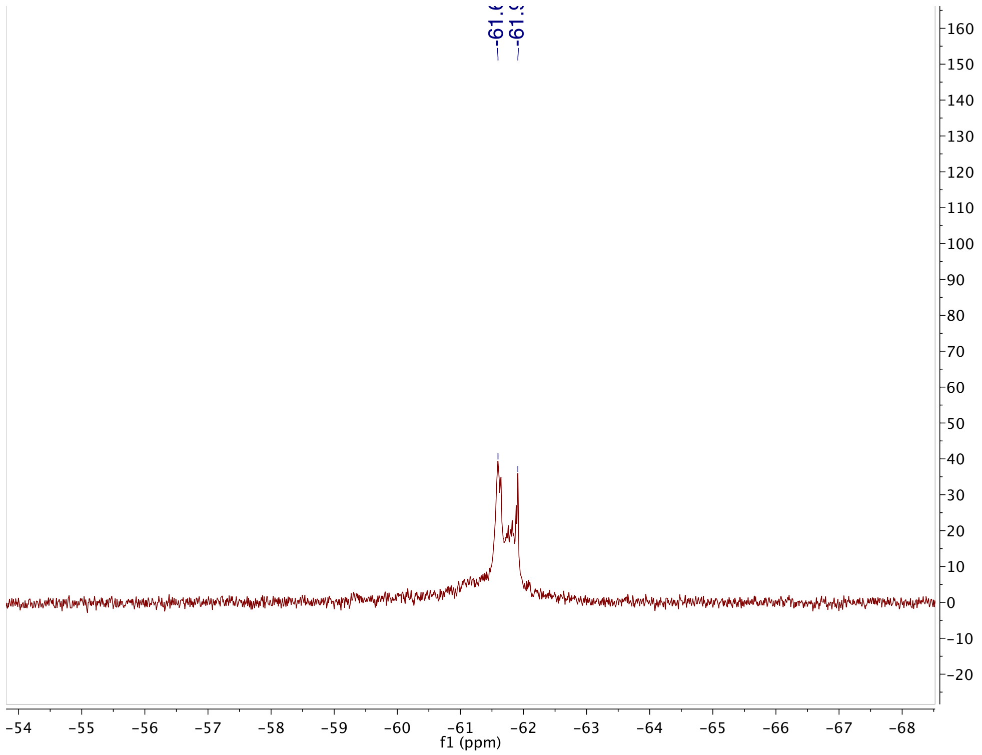

Figure S22: ^{19}F NMR spectrum of **2** and $Cp_2FeBArF_{24}$ in C_6D_6 at 293 K. $BArF_{24}$ is expected to show only one peak and the presence of multiple peaks suggests activation of the $BArF_{24}$ anion.

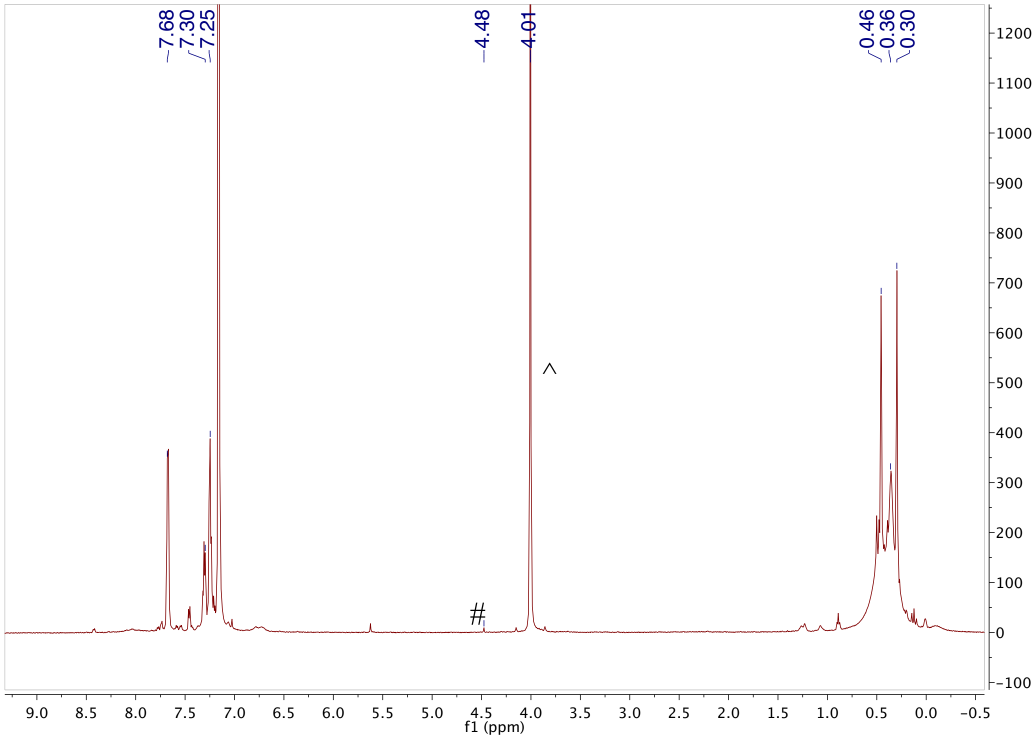

Figure S23: ^{1}H NMR Spectrum of **2** and Cp_2FeBPh_4 in C_6D_6 at 293 K showing the formation of ferrocene (^) and hydrogen (#).

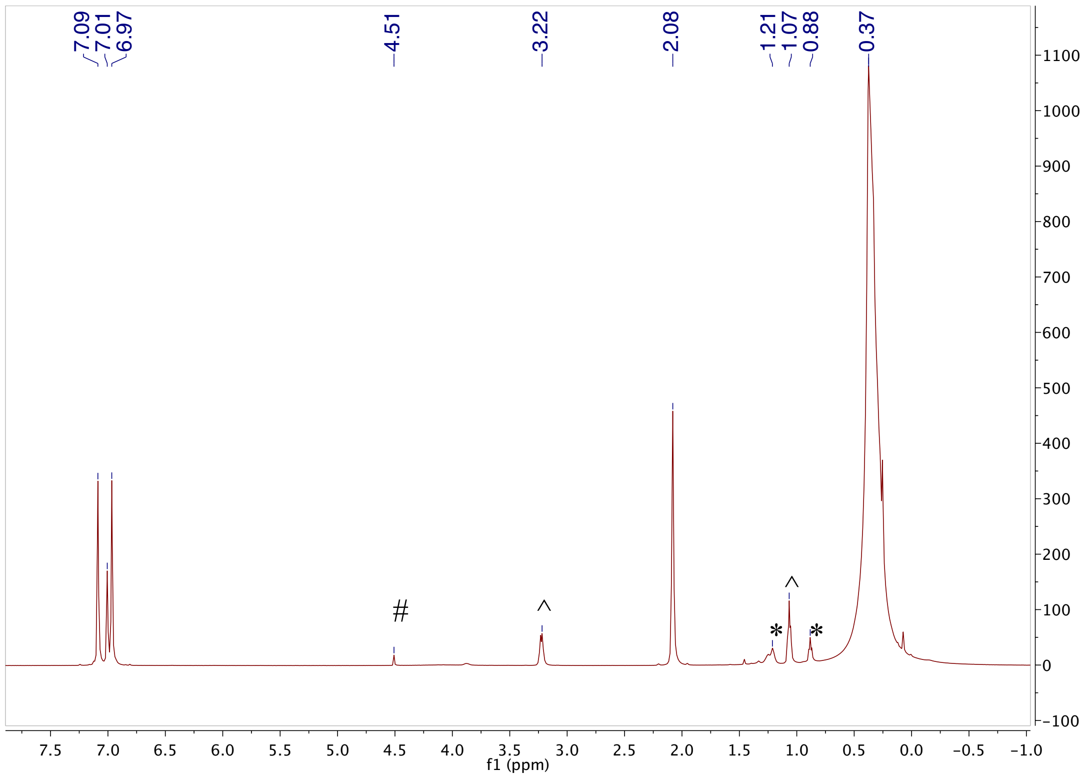

Figure S24: ^{1}H NMR Spectrum of **2** and AgOTf in C_7D_8 at 293 K showing the formation of hydrogen (#). No HD was observed. (^ Et_2O, * hexane)

C. Infrared Spectra

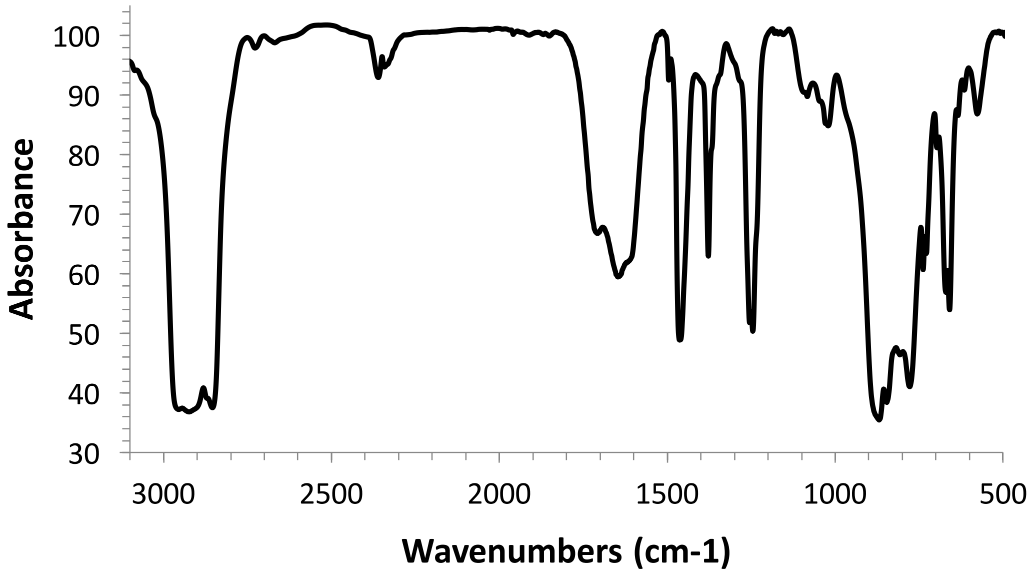

Figure S25: Infrared spectrum of **1**

1647 (s, br), 1245 (s), 1020 (w), 868 (s, br), 777 (m), 728 (m), 670 (m), 657 (m). 576 (w)

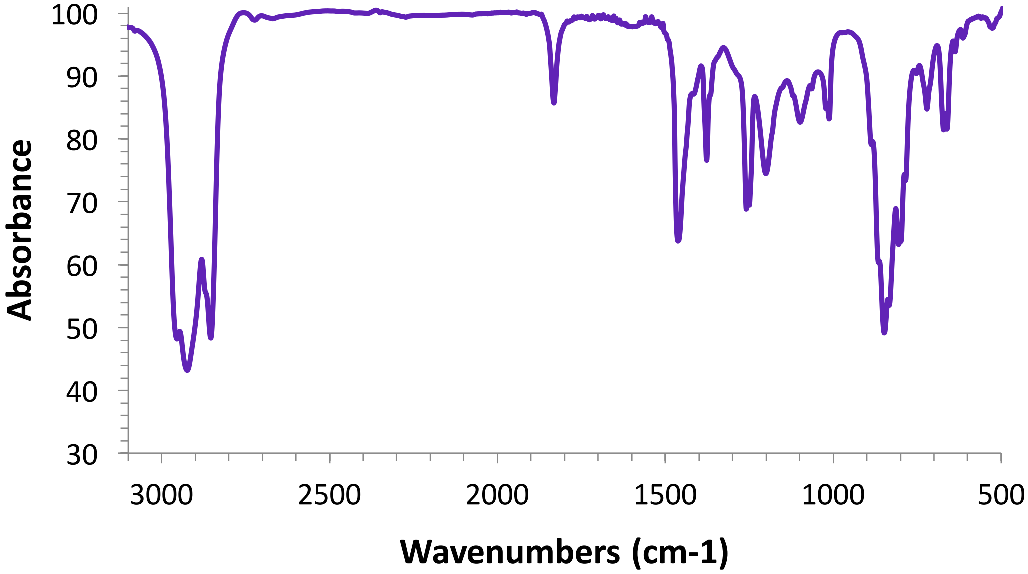

Figure S26: Infrared spectrum of **2**

1831 (m), 1259 (m), 1250 (m), 1201 (m), 1099 (w), 1012 (w), 848 (s, br), 805 (m), 721 (w), 672 (w), 661 (w)

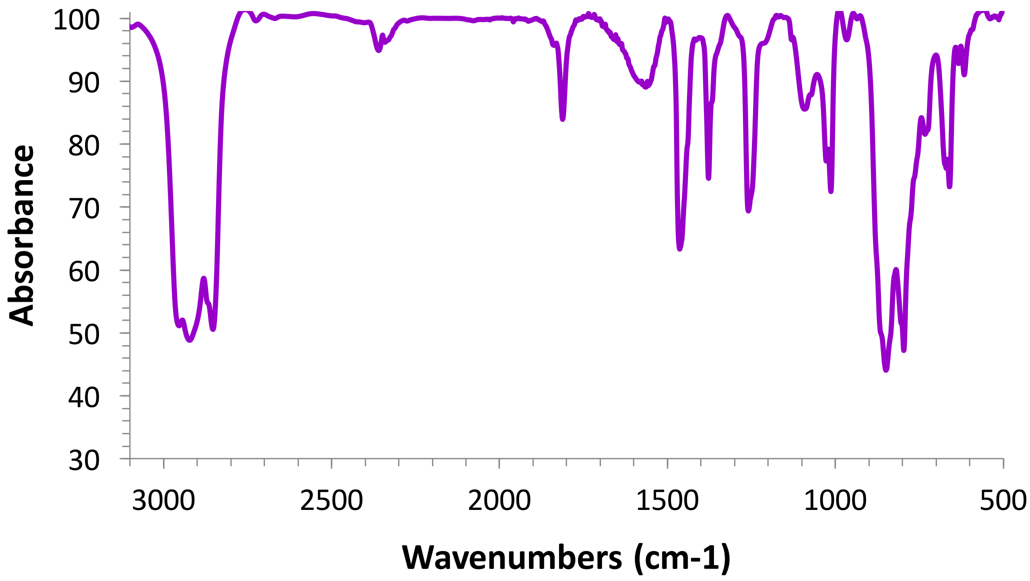

Figure S27: Infrared spectrum of **3**

1811 (m), 1564 (m), 1258 (m), 1091 (w), 1012 (m), 849 (s, br), 795 (s), 732 (w), 659 (m)

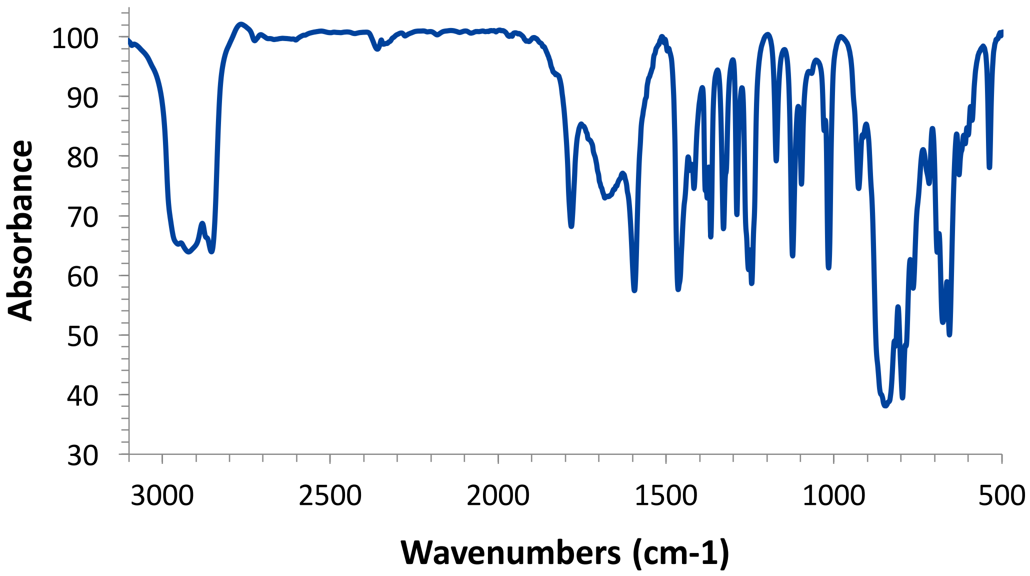

Figure S28: Infrared spectrum of **4**

1783 (m), 1666 (m, br), 1595 (m), 1367 (m), 1330 (w), 1256 (m), 1246 (m), 1172 (w), 1124 (m), 1097 (w), 1017 (m), 927 (w), 846 (s, br), 797 (s), 765 (m), 677 (m), 657 (m)

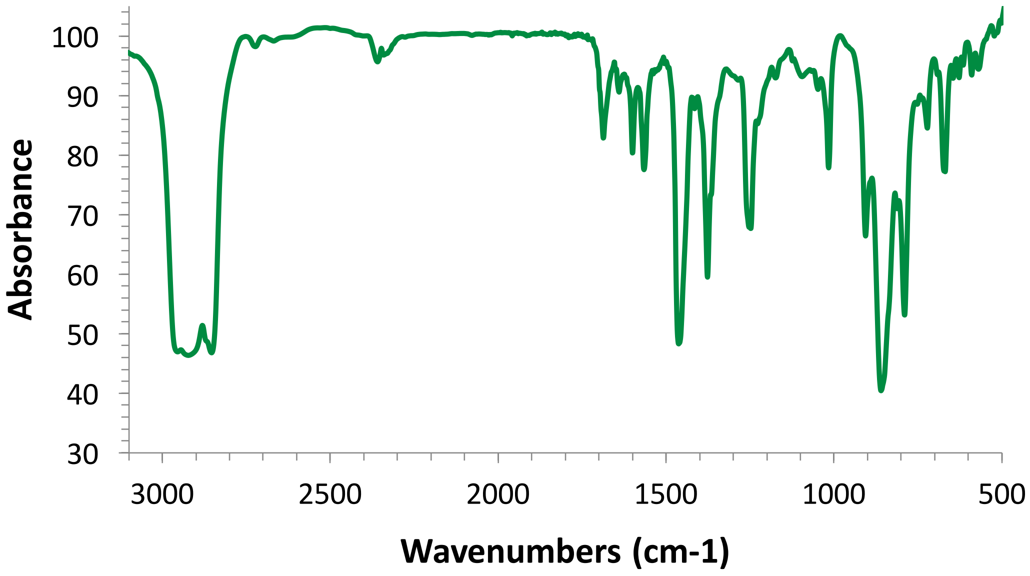

Figure S29: Infrared spectrum of **5**

1687 (m), 1600 (m), 1566 (m), 1248 (m), 1016 (m), 906 (s), 860 (s), 790 (s), 722 (w), 669 (m)

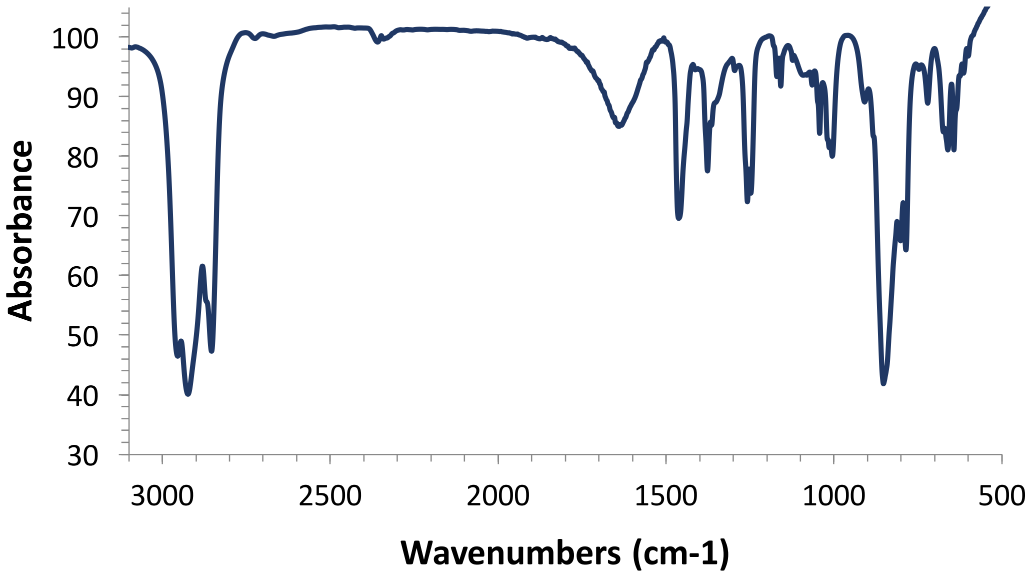

Figure S30: Infrared spectrum of **6**

1640 (m, br), 1258 (m), 1248 (m), 1043 (w), 1005 (w), 853 (s, br), 786 (m), 661 (w), 643 (w)

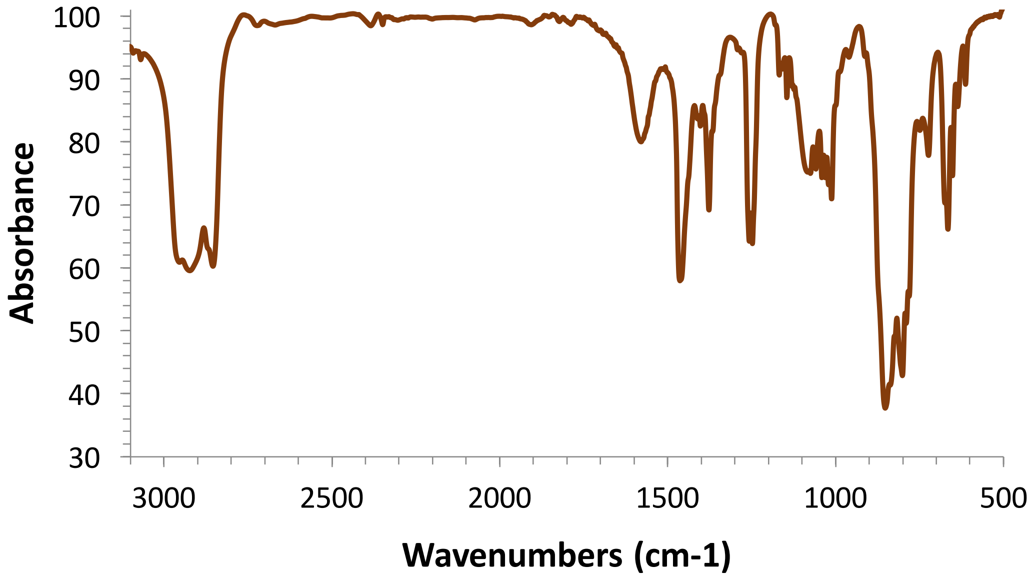

Figure S31: Infrared spectrum of **7**

1579 (m, br), 1257 (m), 1248 (m), 1075 (m, br), 1012 (m), 852 (s), 801 (s), 723 (w), 666 (m)

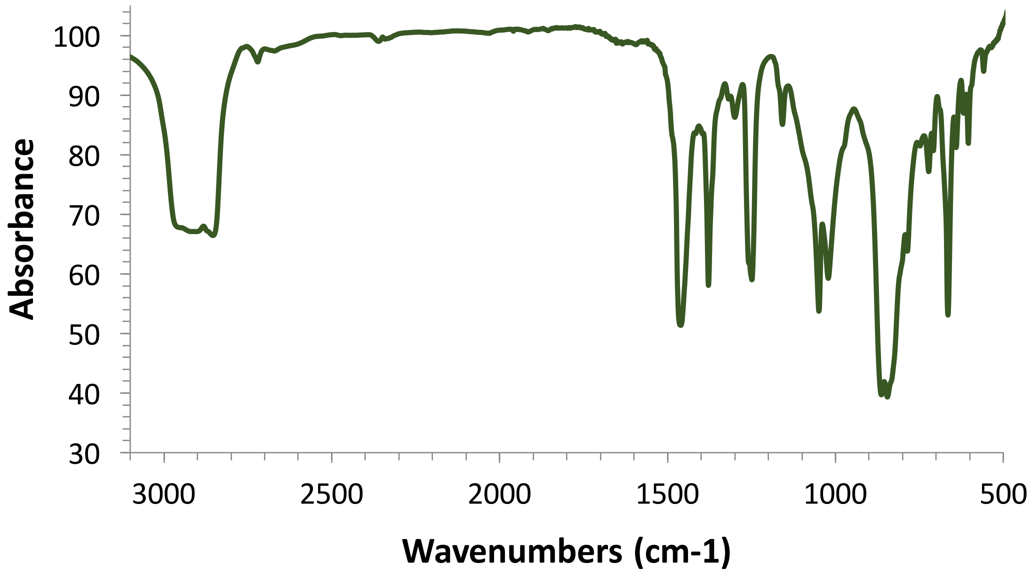

Figure S32: Infrared spectrum of **8**

1248 (m), 1049 (m), 1021 (m), 853 (s, br), 785 (m), 665 (m)

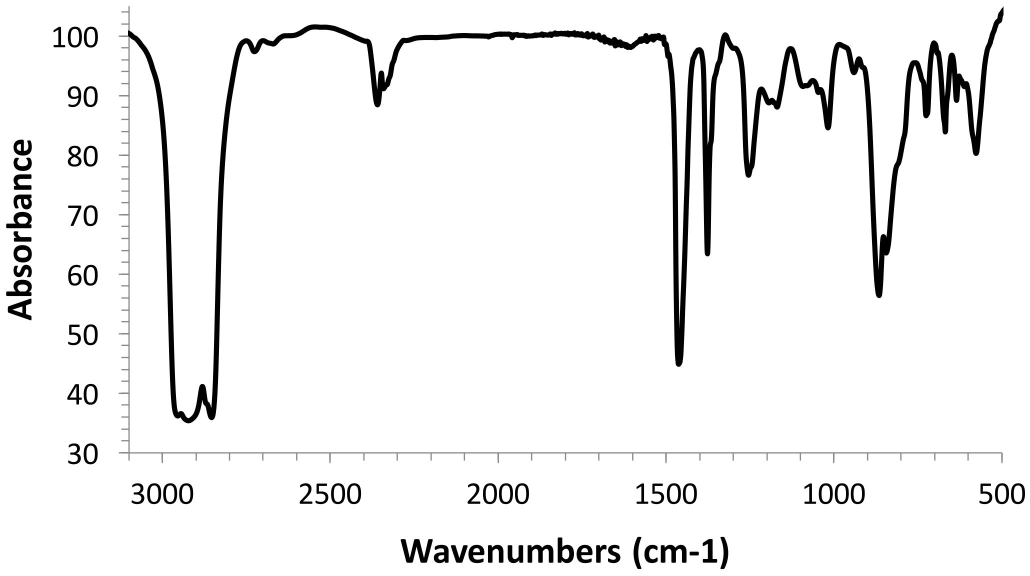

Figure S33: Infrared spectrum of **1D** showing the absence of the strong Al-H stretch at 1646cm^{-1}

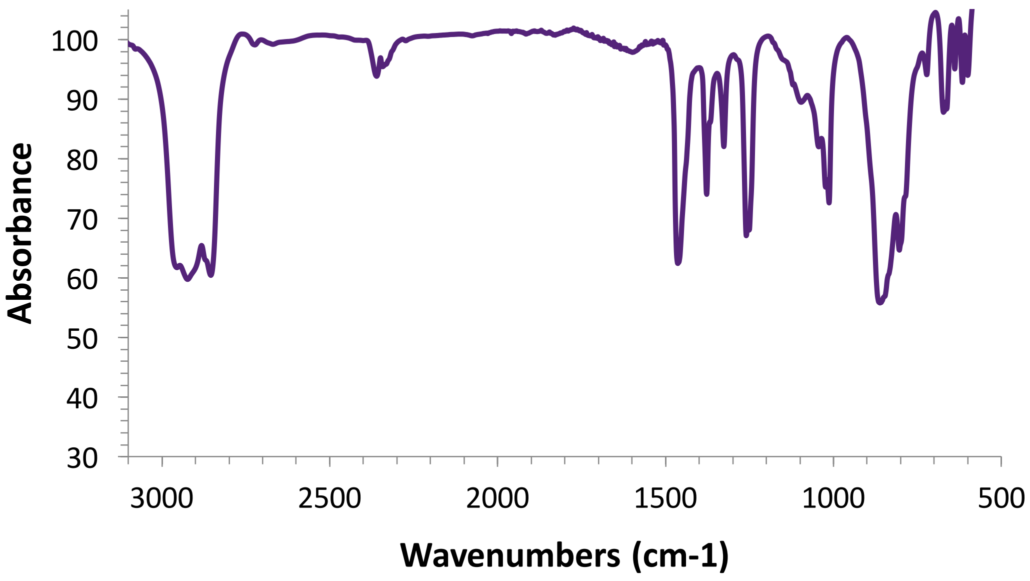

Figure S34: Infrared spectrum of **2D** showing the absence of the Al-H stretch at 1831 cm^{-1}

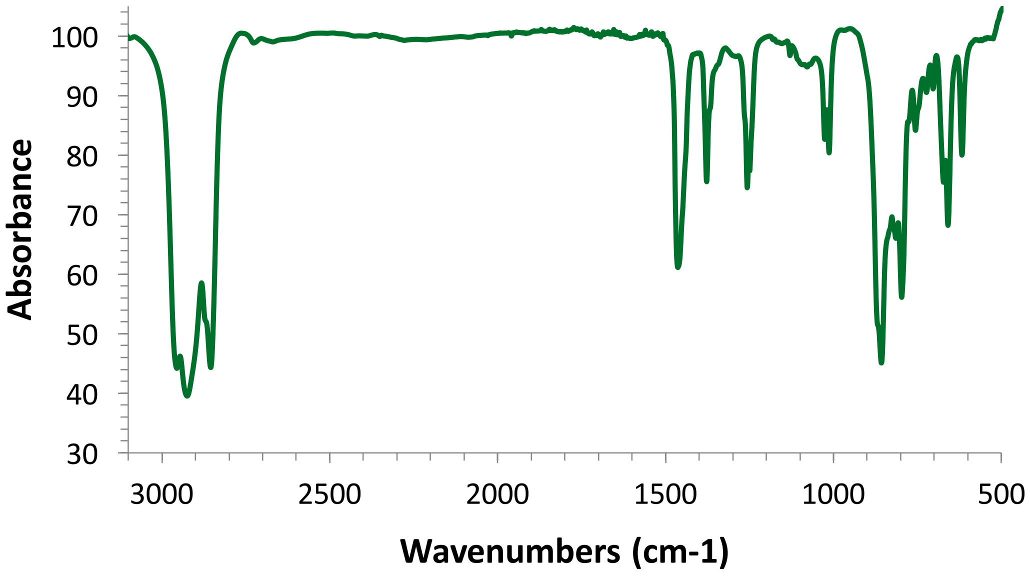

Figure S35: Infrared spectrum of **10**

1256 (m), 1025 (m), 1012 (m), 856 (s), 813 (m), 796 (s), 755 (w), 672 (m), 658 (m), 617 (m)

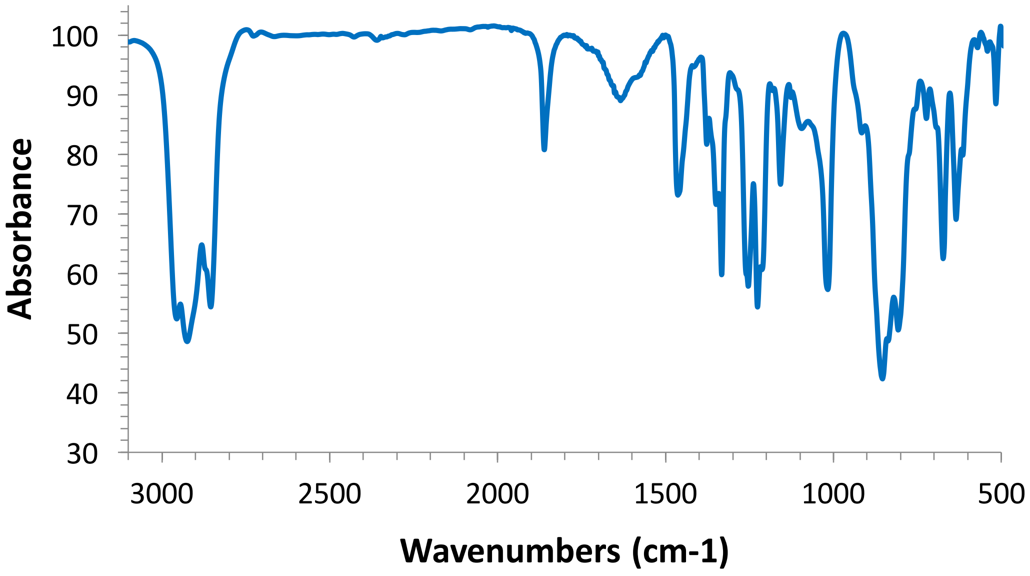

Figure S36: Infrared spectrum of **11**

1860 (m), 1633 (m, br), 1332 (s), 1253 (s), 1226 (s), 1157 (m), 853 (s) 807 (s), 722 (w), 673 (m), 634 (m), 516 (w)

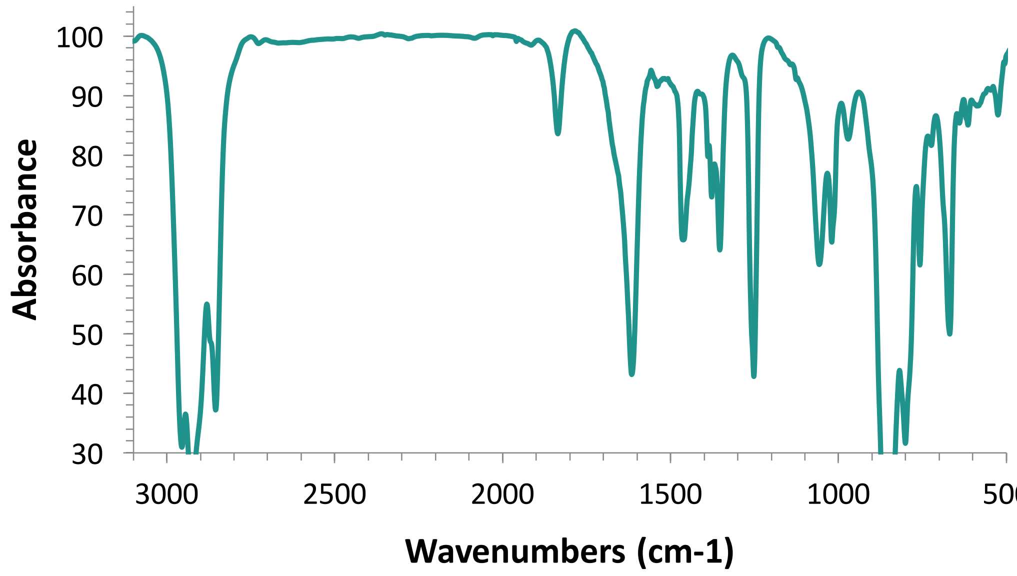

Figure S37: Infrared spectrum of crude product of **3** and CO_2. The strong broad IR stretch at 1615 cm^{-1} suggests a bridging formate moiety.

D. UV-Visible Spectra

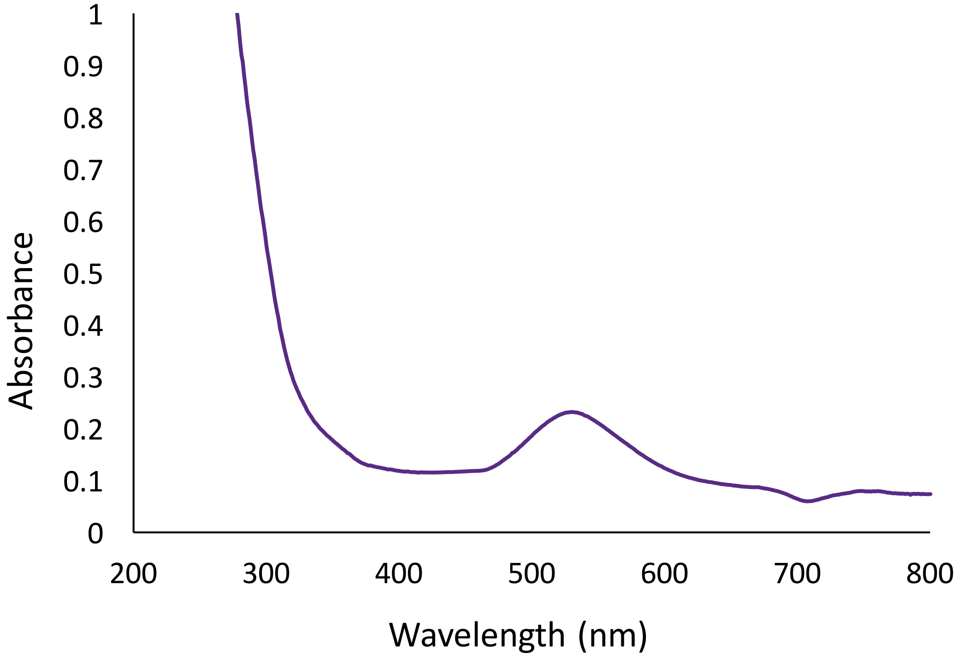

Figure S38: UV-Visible spectrum of 0.077 mM **2** in toluene

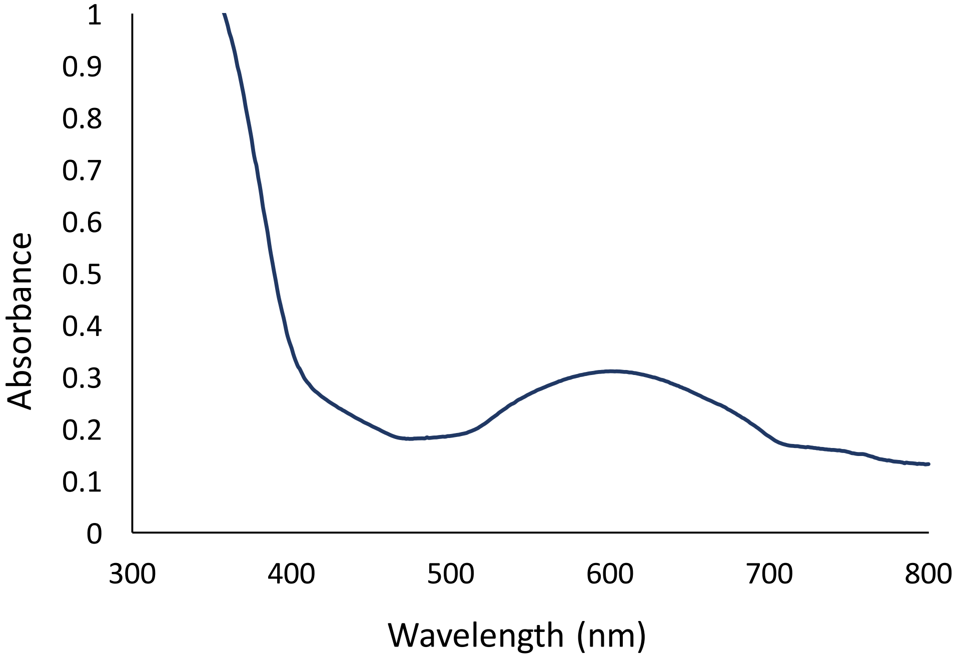

Figure S39: UV-Visible spectrum of 0.495 mM **6** in toluene

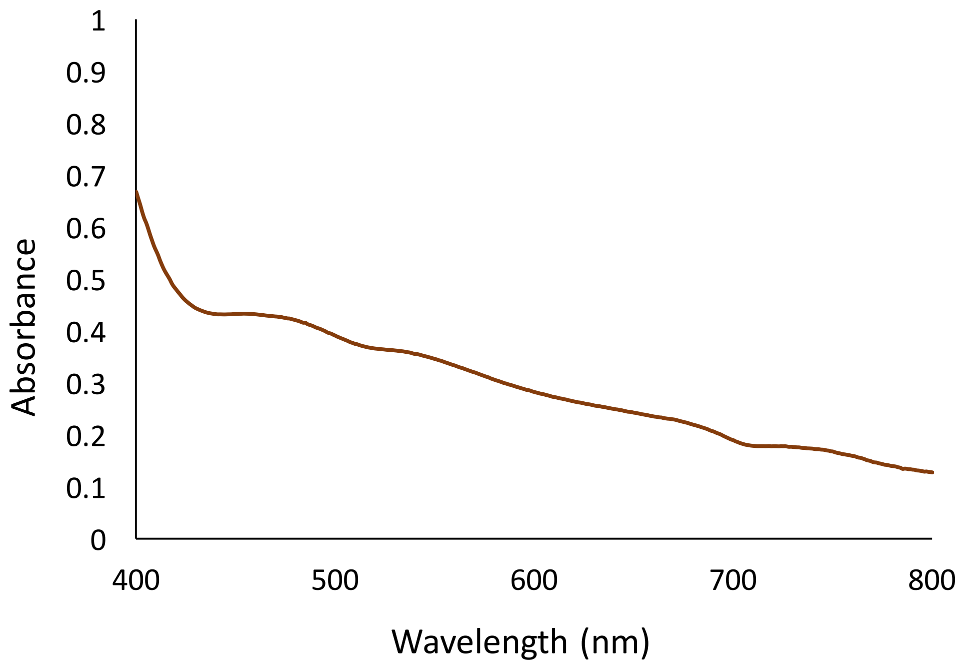

Figure S40: UV-Visible spectrum of 1.6 mM **7** in toluene

E. Cyclic Voltammetry

General considerations: All cyclic voltammograms were recorded in 1,2-difluorobenzene with 0.1 M NBu_4BArF_{24} as a supporting electrolyte

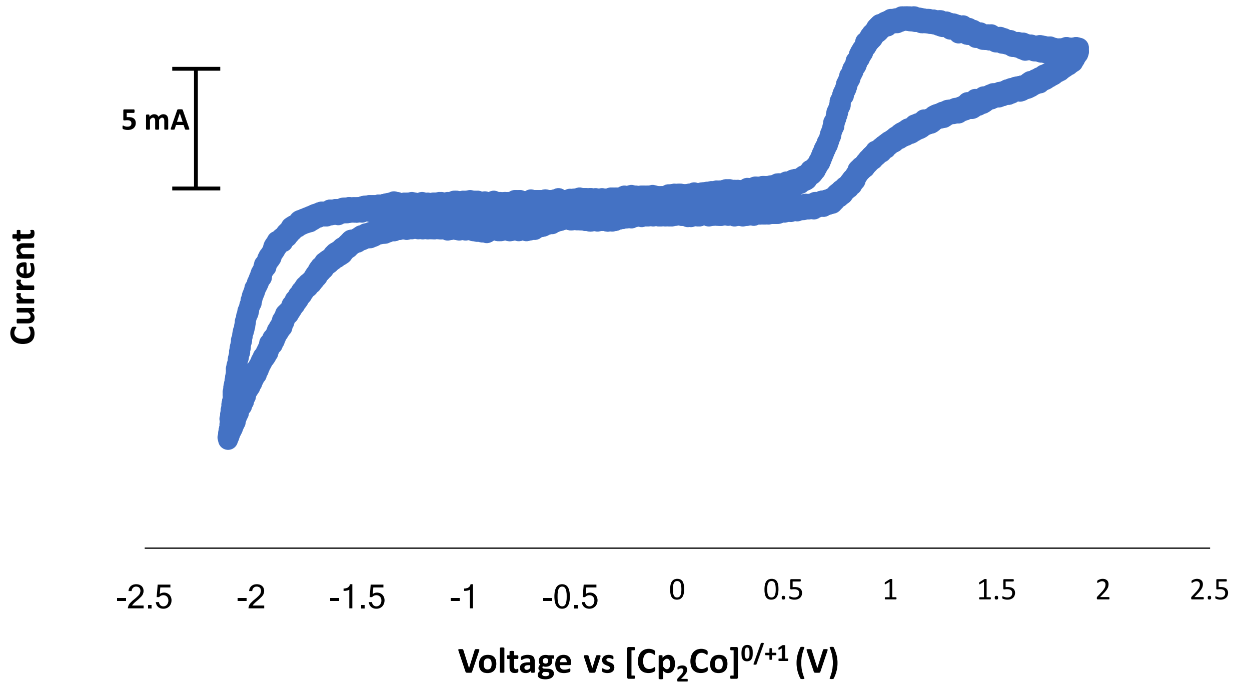

Figure S41: Cyclic Voltammogram of **2** over the full solvent window with a scan rate of rate was 100 mv/s. Once cycle was shown; on the second cycle current was lost due to plating onto the electrode. An irreversible oxidation is visible at 1.13 V vs Cp_2Co/Cp_2Co^+

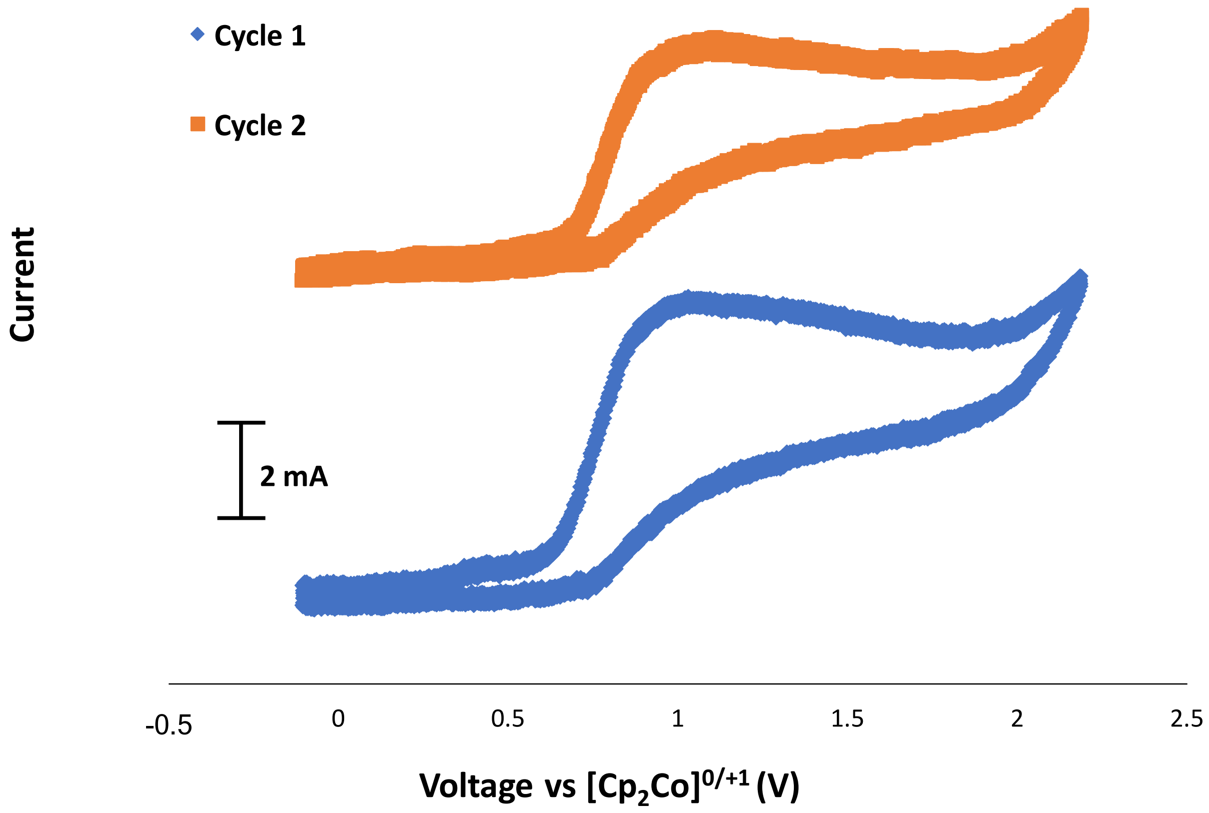

Figure S42: Cyclic Voltammogram of **2** showing only the irreversible oxidation; the scan rate was 100 mv/s. The oxidation does not become reversible when it is studied in isolation. Current decreased on the second cycle due to plating onto the electrode

F. Computational Details

All calculations were carried out with the Gaussian 09 program (G09)[8], employing the B3LYP[9] functional with standard 6-31G+(d,p)[10] basis set to fully optimize the geometries of the complexes. All resultant stationary points were subsequently characterized by vibrational analyses.

Since a test calculation between two intermediates with the $C(TMS)_3$ and CH_3 ligand showed the same relative energies with the full and model system, the abbreviated methyl model ligand was used for all further calculations. Additionally, we studied a unimolecular pathway rather than a bimolecular pathway. Since the same types of bonds are activated in a unimolecular or bimolecular process, the trends in energies determined for a monomeric system are expected to apply to potential bimolecular pathways while simplifying comparison between systems and pathways. While trends in the energies of the transition states are expected to remain the same comparing a unimolecular or bimolecular reaction mechanism, the absolute barriers to activation may not remain the same. For this reason, while the calculated barriers are quite high, the different pathways may still be compared.

For the thermal reactivity of **2'**, two intermediates were calculated. The intermediate corresponding to reductive elimination (**A**) of two hydrides is 14.3 kcal/mol higher in energy than that corresponding to elimination via σ-bond metathesis (**B**). However, the transition states for formation of each intermediate are at similar energies ($\Delta\Delta G^{\ddagger}$ = 7.5 kcal/mol). The small energy difference favors σ-bond metathesis, but it is not significant enough to disregard either pathway given the high reaction temperature (100 °C). The transition state for the oxidative addition of the Cp C-H bond between the two intermediates was also calculated and was found to be 73.3 kcal/mol higher energy than the starting material, suggesting that the oxidative addition of this bond in a unimolecular system is thermally inaccessible (Figure S43). Despite the tendency for early metals to react *via* σ-bond metathesis rather than reductive elimination, the transition states for reductive elimination and σ-bond metathesis in this system are at similar energies and cannot be differentiated with DFT calculations.

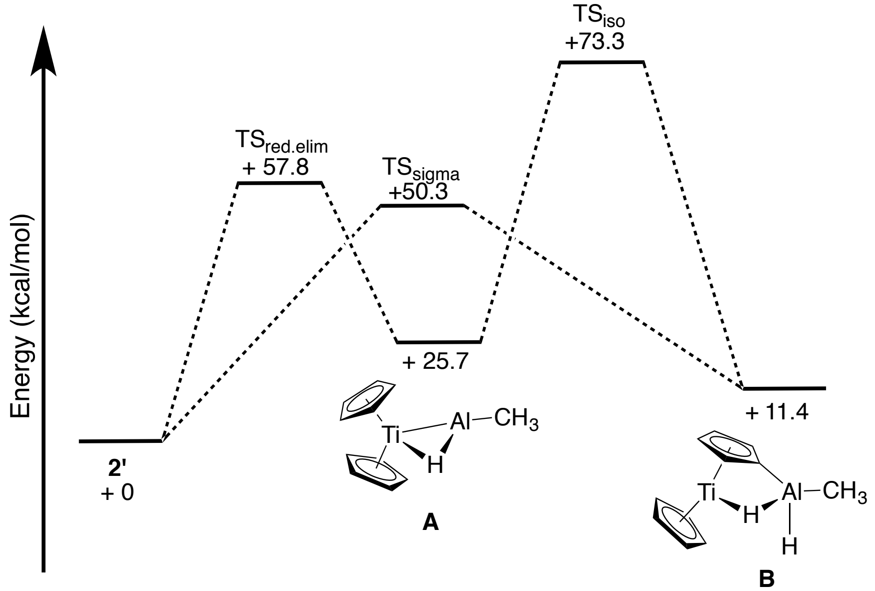

Figure S43: DFT Calculated reaction scheme for the conversion of **2'** to **A** and **B**

Cartesian coordinates of the optimized complexes:

Computational Model for **2, 2’**

Ti	0.274141	0.039940	-0.003316
Al	-2.058951	-1.454245	0.001301
C	0.129916	2.097618	1.185155
H	0.374636	2.140089	2.238130
C	-1.159102	1.869029	0.634354
H	-2.065157	1.688830	1.195315
C	1.363397	-1.947556	0.833446
H	0.881429	-2.565111	1.578378
C	-1.050576	1.906981	-0.780210
H	-1.858297	1.753703	-1.482217
C	0.305163	2.153087	-1.113469
H	0.709395	2.243456	-2.113009
C	2.615101	-0.308946	-0.170835
H	3.290045	0.521690	-0.320688
C	2.208709	-0.839293	1.086952
H	2.505379	-0.473300	2.061110
C	1.030804	2.286026	0.103528
H	2.083961	2.511410	0.192166
C	1.229866	-2.090119	-0.572388
H	0.635075	-2.839487	-1.074973
C	2.002539	-1.074333	-1.197566
H	2.115036	-0.924510	-2.263091
H	-0.998926	-0.743582	1.141051
H	-1.007098	-0.738541	-1.143279
H	-1.885201	-3.039438	0.008239
C	-3.883200	-0.692483	-0.001619
H	-3.914509	0.400864	-0.072409
H	-4.463325	-1.091820	-0.844175

H	-4.422876	-0.977805	0.911404

Computational Model for **6**

C	-1.460048	1.099803	1.719254
C	-2.726575	1.476138	1.155635
H	-2.931236	2.399695	0.627288
C	-3.663560	0.438992	1.347248
H	-4.692742	0.434441	1.010484
C	-3.009798	-0.604561	2.062005
H	-3.457243	-1.541193	2.370137
C	-1.676603	-0.199435	2.294021
H	-0.933679	-0.792108	2.811876
C	-1.360968	-2.312004	-1.471283
H	-0.343362	-2.659545	-1.576386
C	-2.278451	-2.720902	-0.471260
H	-2.097102	-3.459313	0.298697
C	-3.493467	-2.010321	-0.681667
H	-4.396383	-2.113551	-0.096141
C	-3.318143	-1.145876	-1.791602
H	-4.057559	-0.471393	-2.202528
C	-1.996143	-1.339027	-2.280737
H	-1.556640	-0.827323	-3.125967
C	1.460215	1.098988	-1.719578
C	1.676572	-0.200504	-2.293839
H	0.933563	-0.793225	-2.811512
C	3.009691	-0.605771	-2.061624
H	3.456988	-1.542601	-2.369372
C	3.663615	0.437965	-1.347284
H	4.692792	0.433383	-1.010507
C	2.726800	1.475349	-1.156110
H	2.931611	2.399093	-0.628148

C	1.995965	-1.338150	2.281164
H	1.556499	-0.826167	3.126246
C	1.360865	-2.311524	1.472131
H	0.343287	-2.659103	1.577380
C	2.278370	-2.720751	0.472264
H	2.097076	-3.459503	-0.297379
C	3.493348	-2.010020	0.682398
H	4.396270	-2.113432	0.096915
C	3.317953	-1.145113	1.791962
H	4.057293	-0.470359	2.202578
Al	-0.232593	2.083193	-1.521139
Al	0.232876	2.083708	1.520411
Ti	-1.860885	-0.392951	-0.056345
Ti	1.860648	-0.393040	0.056334
H	-1.486591	1.044241	-1.215583
H	1.486808	1.044575	1.215183
H	0.000124	2.848733	-0.000494
H	-0.000129	-0.560364	0.000146
C	0.857103	3.495637	2.750910
H	1.776268	3.977606	2.395538
H	0.099644	4.279733	2.876205
H	1.067359	3.086837	3.748078
C	-0.856659	3.494718	-2.752181
H	-1.775306	3.977500	-2.396572
H	-0.098785	4.278253	-2.878465
H	-1.067789	3.085382	-3.748944

Sigma Bond Metathesis intermediate

Ti	0.314649	-0.048247	-0.000090
Al	-2.198268	-0.607863	-0.000839
C	1.607188	-1.946762	-0.709309

H	1.164063	-2.702716	-1.343145
C	1.606901	-1.946842	0.709317
H	1.163260	-2.702678	1.342929
C	-0.770621	1.693307	-1.143525
H	-1.033988	1.513547	-2.179136
C	2.261066	-0.767221	1.153504
H	2.426389	-0.479033	2.183234
C	2.672657	-0.042266	0.000348
H	3.211255	0.895681	0.000461
C	0.431707	2.288382	0.714311
H	1.215739	2.686018	1.347660
C	0.432247	2.288587	-0.713444
H	1.216770	2.686412	-1.346063
C	2.261527	-0.767089	-1.153124
H	2.427747	-0.479111	-2.182765
C	-1.543171	1.263790	-0.000466
C	-0.771490	1.692980	1.143296
H	-1.035625	1.512894	2.178653
H	-0.951644	-1.015424	-1.119193
H	-0.951885	-1.015503	1.118103
C	-3.918781	-1.546580	0.000582
H	-3.786632	-2.634387	0.001613
H	-4.512125	-1.280984	0.883668
H	-4.513498	-1.282767	-0.882092

Reductive Elimination Intermediate

Ti	-0.252392	0.008704	0.020068
Al	2.239652	-0.327775	-0.345643
C	-1.680296	-1.561541	1.109507

H	-2.085618	-1.347540	2.089416
C	-0.478062	-2.258438	0.839541
H	0.198235	-2.661415	1.581086
C	0.119133	2.193579	0.986678
H	0.743065	2.320568	1.860587
C	-0.308148	-2.327647	-0.574106
H	0.475488	-2.854399	-1.099407
C	-1.417294	-1.672609	-1.180768
H	-1.592556	-1.572045	-2.243459
C	-1.701246	1.851657	-0.368152
H	-2.717338	1.688136	-0.699653
C	-1.270259	1.938083	0.989740
H	-1.898023	1.841668	1.865500
C	-2.267491	-1.213242	-0.139649
H	-3.209985	-0.700266	-0.271775
C	0.560753	2.232596	-0.370589
H	1.548066	2.516056	-0.710261
C	-0.577264	2.024905	-1.208713
H	-0.576601	2.018414	-2.290184
H	1.231593	-0.233505	1.123831
C	4.145070	-0.456420	0.218201
H	4.308316	-1.360086	0.821622
H	4.837474	-0.492817	-0.630376
H	4.425661	0.401285	0.844799

H_2

H	0.000000	0.000000	0.371394
H	0.000000	0.000000	-0.371394

Sigma Bond Metathesis Transition State

Ti	0.336290	-0.028997	-0.027096

Al	-2.189409	-0.624842	-0.320863
C	2.305436	-0.794592	-1.113039
H	2.543926	-0.468603	-2.116885
C	1.564019	-1.954861	-0.764060
H	1.123402	-2.657273	-1.458360
C	-0.473775	1.862060	-1.114917
H	-0.615871	1.809903	-2.188123
C	1.485664	-2.023339	0.651608
H	0.975376	-2.786267	1.222798
C	2.177769	-0.905210	1.186916
H	2.300549	-0.675713	2.237149
C	0.452410	2.215696	0.954799
H	1.158322	2.490069	1.729763
C	0.673997	2.333180	-0.454286
H	1.567696	2.728926	-0.920668
C	2.692385	-0.151625	0.095025
H	3.281023	0.752638	0.171592
C	-1.428230	1.402510	-0.130754
H	-2.584609	0.998742	-0.901733
C	-0.835748	1.686675	1.152424
H	-1.270155	1.443376	2.113681
H	-0.848043	-0.882740	-1.313751
H	-0.909690	-0.934382	0.927898
H	-3.089986	0.352142	-1.553750
C	-3.701493	-1.649850	0.400235
H	-4.234995	-2.187310	-0.392242
H	-3.381180	-2.374532	1.156923
H	-4.426437	-0.971963	0.869354

Reductive Elimination Transition State

Ti	-0.293712	0.022202	-0.043043

Al	2.113679	-1.036028	-0.016189
C	-0.647279	2.235641	-0.900871
H	-1.307022	2.394498	-1.743636
C	0.745224	2.011136	-0.966685
H	1.336369	1.962289	-1.871506
C	-1.213916	-2.134148	-0.575080
H	-0.611353	-2.875001	-1.083156
C	1.231246	1.828095	0.363141
H	2.268560	1.745138	0.656903
C	0.125101	1.969440	1.254253
H	0.166257	1.905041	2.333285
C	-2.625804	-0.375034	-0.176037
H	-3.303982	0.454058	-0.325548
C	-2.013335	-1.139988	-1.202222
H	-2.138106	-1.001444	-2.267967
C	-1.026642	2.225240	0.472831
H	-2.026211	2.381934	0.855010
C	-1.335467	-1.986073	0.833652
H	-0.883967	-2.621195	1.583132
C	-2.197321	-0.887033	1.081864
H	-2.490900	-0.516449	2.055271
H	1.067714	-0.768514	-1.276912
H	1.125433	-1.985840	0.883809
H	1.681047	-2.724612	0.361786
C	4.073571	-0.881630	-0.015279
H	4.486179	-1.339226	0.891390
H	4.522886	-1.389679	-0.874723
H	4.398649	0.165054	-0.037147

Isomerization Transition State

Ti	-0.332020	0.007802	0.038751

Al	2.077228	-0.972379	-0.167467
C	-2.332577	-0.729430	1.117926
H	-2.594346	-0.379398	2.107700
C	-1.583943	-1.893703	0.813308
H	-1.159177	-2.579418	1.534441
C	1.202524	1.605420	0.836672
H	1.911480	1.407247	1.630740
C	-1.467597	-1.993664	-0.601022
H	-0.960249	-2.778499	-1.144531
C	-2.149588	-0.887421	-1.177332
H	-2.255335	-0.684136	-2.234675
C	-0.677606	2.343979	-0.237879
H	-1.647248	2.787623	-0.427279
C	-0.090133	2.154753	1.044244
H	-0.525124	2.434298	1.995725
C	-2.686532	-0.109946	-0.113363
H	-3.276927	0.789578	-0.220944
C	0.233423	1.876500	-1.214786
H	0.045116	1.847598	-2.281913
H	0.979138	-0.904145	1.104634
C	3.982318	-1.345707	0.107217
H	4.385342	-0.744468	0.929856
H	4.151379	-2.401534	0.346405
H	4.554501	-1.107029	-0.795534
C	1.460851	1.425807	-0.578926
H	1.345091	0.102028	-1.196420

G. Crystallographic Details

	1	2	3	4	5
Chemical formula	$C_{18}H_{46}AlKO_2Si_3$	$C_{20}H_{40}AlSi_3Ti$	$C_{30}H_{51}AlSi_3Ti_2$	$C_{27}H_{54}AlN_2Si_3Ti$	$C_{30}H_{56}AlN_2O_3Si_3Ti$
Formula weight	444.90	439.67	618.75	565.87	651.91
Color, habit	Colorless, plates	Purple, block	Purple, needles	Blue, blocks	Green, needles
Temperature (K)	100(2)	100(2)	100(2)	100(2)	100(2)
Crystal system	Triclinic	Monoclinic	Triclinic	Monoclinic	Orthorhombic
Space group	P -1	P 2_1/c	P -1	P 2_1/n	Pna2_1
a (Å)	13.5985(5)	18.6834(11)	11.8167(11)	11.686(8)	12.528(7)
b (Å)	13.6009(5)	9.2143(5)	16.7464(16)	15.467(10)	18.992(10)
c (Å)	16.6858(7)	15.3519(8)	18.0477(18)	18.639(12)	15.352(8)
α (°)	106.549(2)	90	105.953(4)	90	90
β (°)	107.020(2)	108.428(2)	103.150(4)	107.884(5)	90
γ (°)	98.456(2)	90	90.965(4)	90	90
V ($Å^3$)	2737.83(19)	2507.4(2)	3331.8(6)	3212(4)	3653(3)
Z	4	4	4	4	4
Densitiy (Mg m^{-3})	1.079	1.165	1.234	1.170	1.185
F(000)	976	948	1320.0	1228	1404
Radiation Type	MoK_α	MoK_α	MoK_α	Synchrotron	Synchrotron
μ (mm^{-1})		0.522	0.631	0.495	0.468
Crystal size	0.18 x 0.16 x 0.16	0.20 x 0.20 x 0.20	0.16 x 0.06 x 0.04	0.6 x 0.4 x 0.16	0.15 x 0.05 x 0.05
Meas. Refl.	15870	62302	148243	32982	36396
Indep. Refl.	8712	4606	12286	7486	8342
R(int)	0.0402	0.0466	0.0738	0.390	0.0499
Final R indices [I > 2σ(I)]	R = 0.0437<br>R_w = 0.1307	R = 0.0418<br>R_w = 0.1113	R = 0.0933<br>R_w = 0.2365	R = 0.0306<br>R_w = 0.0735	R = 0.0278<br>R_w = 0.0630
Goodness-of-fit	1.024	1.101	1.052	1.041	1.037
$\Delta\rho_{max}$, $\Delta\rho_{min}$ (e $Å^{-3}$)		0.760, -0.532	3.734, -0.976	0.346, -0.404	0.195, -0.313
CCDC	1530362	1530361	1530366	1530368	1530367

	**6**	**7**	**8**	**9**	**10**
Chemical formula	$C_{40}H_{76}Al_2Si_6Ti_2$	$C_{30}H_{47}AlSi_3Ti_2$	$C_{30}H_{58}AlSi_3Ti$	$C_{30}H_{47}AlF_4Si_3Ti_2$	$C_{21}H_{39}AlF_3O_3SSi_3Ti$
Formula weight	875.28	614.72	577.91	690.72	587.73
Color, habit	Blue, block	Brown, needles	Green, blocks	Green, blocks	Blue, plates
Temperature (K)	100(2)	100(2)	100(2)	100(2)	100(2)
Crystal system	Monoclinic	Monoclinic	Monoclinic	Triclinic	Monoclinic
Space group	P 2_1/c	P 2_1/c	P 2_1/c	P -1	P 2_1/n
a (Å)	13.8040(5)	13.9593(11)	17.5367(5)	12.1490(6)	15.9334(5)
b (Å)	20.5426(10)	26.812(2)	11.3812(3)	16.6519(9)	8.8807(2)
c (Å)	18.7994(9)	9.5669(6)	18.6758(5)	18.1791(9)	41.2358(12)
α (°)	90	90	90	105.807(3)	90
β (°)	105.291(2)	90.391(4)	115.366(1)	102.060(2)	92.756(2)
γ (°)	90	90	90	91.054	90
V ($Å^3$)	5142.2(4)	3580.6(5)	3368.11(16)	3449.4(3)	5828.1(3)
Z	4	4	4	4	8
Densitiy (Mg m^{-3})	1.186	1.140	1.140	1.330	1.340
F(000)	1980.0	1304.0	1260.0	1448.0	2472.0
Radiation Type	MoK_α	MoK_α	MoK_α	MoK_α	MoK_α
μ (mm^{-1})	0.512	0.587	0.404	0.633	0.559
Crystal size	0.30 x 0.20 x 0.20	0.60 x 0.40 x 0.10	0.14 x 0.10 x 0.09	0.08 x 0.06 x 0.05	0.08 x 0.05 x 0.01
Meas. Refl.	127502	73258	44586	113817	127278
Indep. Refl.	9445	6541	6166	12679	10668
R(int)	0.0941	0.1742	0.0395	0.1061	0.0798
Final R indices [I > 2σ(I)]	R = 0.0384 R_w = 0.0896	R = 0.0484 R_w = 0.1128	R = 0.0477 R_w = 0.1232	R = 0.0578 R_w = 0.1261	R = 0.0532 R_w = 0.1174
Goodness-of-fit	1.066	0.945	1.066	1.042	1.038
$\Delta\rho_{max}$, $\Delta\rho_{min}$ (e $Å^{-3}$)	0.529, -0.333	0.937, -0.409	1.450, -0.291	1.452, -0.408	0.616, -0.359
CCDC	1530364	1530360	1530363	1530365	1530369

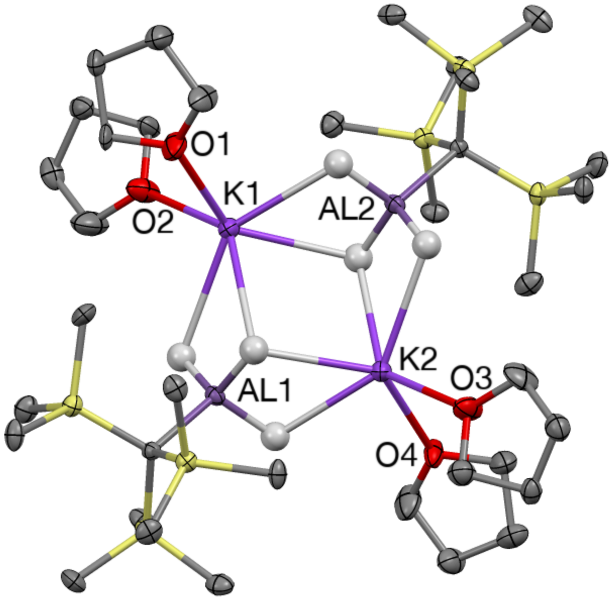

Figure S44: Crystallographically determined structure of **1**. Thermal ellipsoids are shown at the 50% probability level, C-H hydrogen atoms are omitted for clarity. Metal hydrides were located in the Fourier difference map.

H. ESI Mass-Spec Data

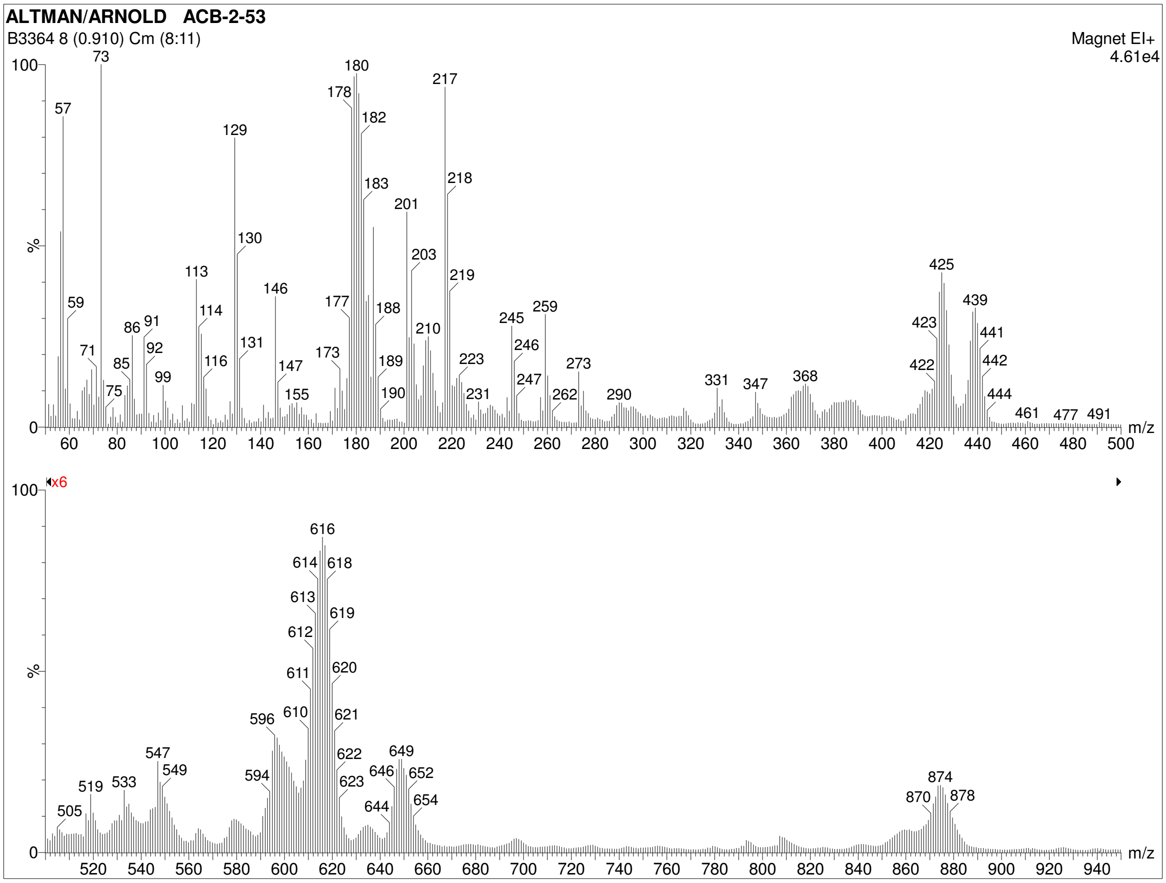

Figure S45: EI-MS data for **6D** showing a broad molecular ion peak around 874 indicative of isotopic scrambling.

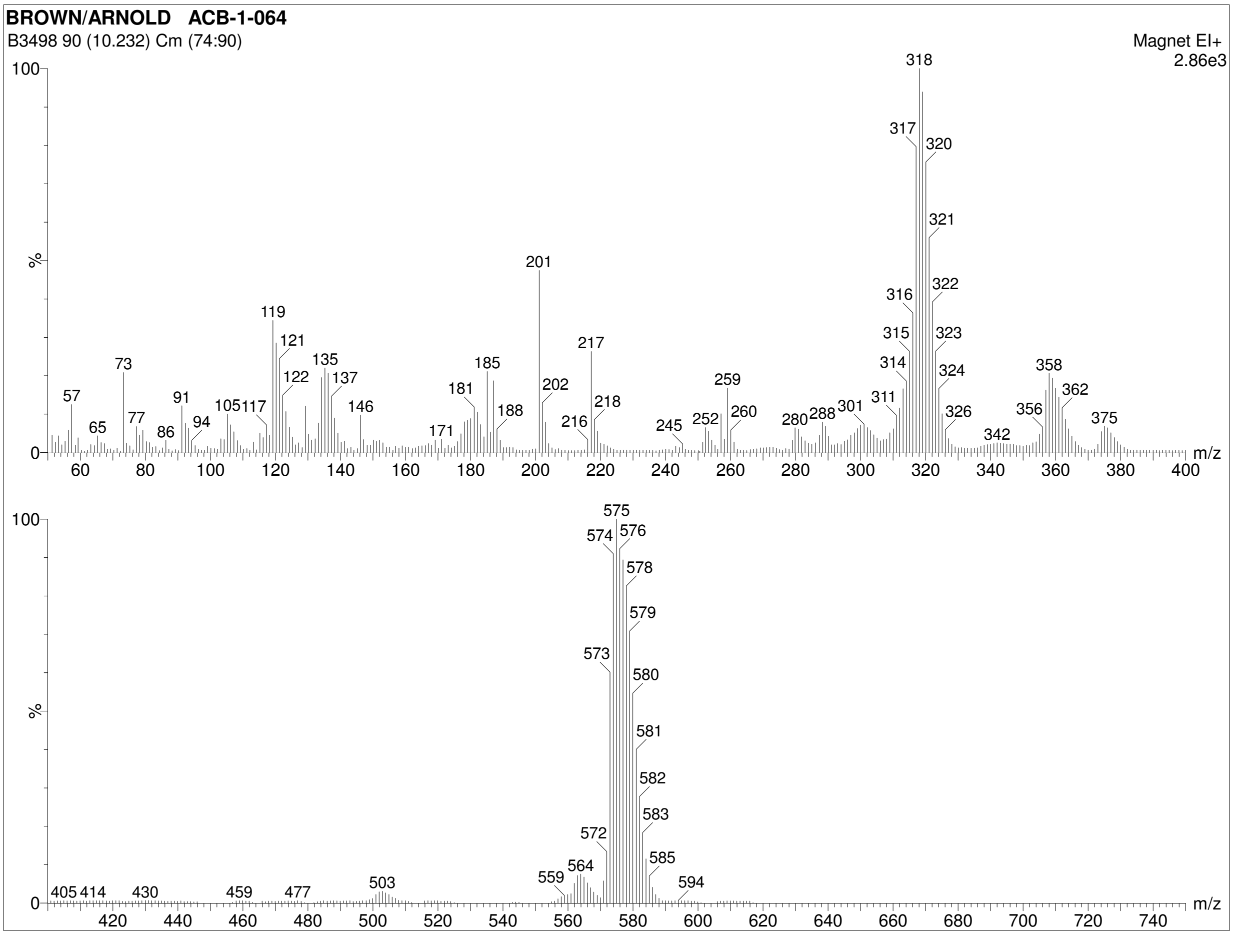

Figure S46: EI-MS data for **8D** showing a broad molecular ion peak around 575 indicative of isotopic scrambling.

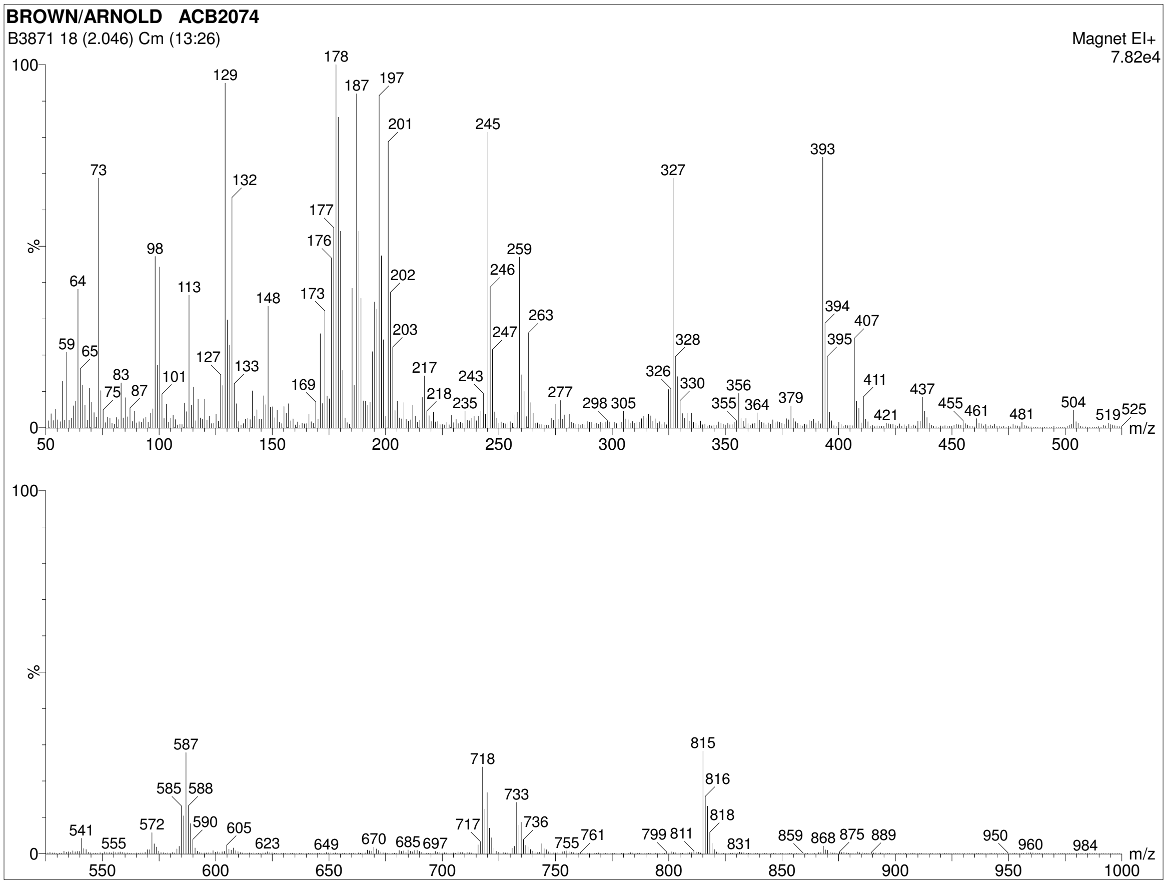

Figure S47: EI-MS data for **10** showing a molecular ion peak around 587. High-resolution mass spectrometry shown a molecular ion with masses of 587.1174, 588.1182 and 589.1154, which fit to a sample with the formula $C_{21}H_{39}O_3F_3AlSi_3STi$ (587.1175) indicating that both metal hydrides are hydrogen and not deuterium. The peaks at +1 and +2 are due to natural abundance of carbon and titanium isotopes.

I. References

1 E. Y.-J. Min, J. E. Bercaw, W. Bernskoetter and P. J. Chirik, in *Inorganic Syntheses, Volume 36*, eds. G. S. Girolami and A. P. Sattelberger, John Wiley & Sons, Inc., Hoboken, NJ, USA, 2014, pp. 47–51.

2 R. P. Spencer and J. Schwartz, *Tetrahedron*, 2000, **56**, 2103–2112.

3 R. S. P. Coutts, P. C. Wailes and R. L. Martin, *J. Organomet. Chem.*, 1973, **47**, 375–382.

4 A. H. Cowley, N. C. Norman, M. Pakulski, G. Becker, M. Layh, E. Kirchner and M. Schmidt, *Inorg. Synth. Vol. 27*, 1990, 235–240.

5 C. Eaborn, I. B. Gorrell, P. B. Hitchcock, J. D. Smith and K. Tavakkoli, *Organometallics*, 1994, **13**, 4143–4144.

6 N. G. Connelly and W. E. Geiger, *Chem. Rev.*, 1996, **96**, 877–910.

7 N. A. Yakelis and R. G. Bergman, *Organometallics*, 2005, **24**, 3579–3581.

8 M. . Frisch, M.; Trucks, G.; Schlegel, H.; Scuseria, G.; Robb, G. . Cheeseman, J.; Scalmani, G.; Barone, V.; Mennucci, B.; Petersson, A. . B. Nakatsuji, H.; Caricato, M.; Li, X.; Hratchian, H.; Izmaylov, K. . J.; Zheng, G.; Sonnenberg, J.; Hada, M.; Ehara, M.; Toyota, Y. . K. Fukuda, R.; Hasegawa, J.; Ishida, M.; Nakajima, T.; Honda, F. . O.; Nakai, H.; Vreven, T.; Montgomery, J.; Peralta, J.; Ogliaro, V. . Bearpark, M.; Heyd, J.; Brothers, E.; Kudin, K.; Staroverov, J. . Kobayashi, R.; Normand, J.; Raghavachari, K.; Rendell, A.; Burant, M. . Iyengar, S.; Tomasi, J.; Cossi, M.; Rega, N.; Millam, J.; Klene, R. . Knox, J.; Cross, J.; Bakken, V.; Adamo, C.; Jaramillo, J.; Gomperts, C. . Stratmann, R.; Yazyev, O.; Austin, A.; Cammi, R.; Pomelli, G. . Ochterski, J.; Martin, R.; Morokuma, K.; Zakrzewski, V.; Voth, A. . F. Salvador, P.; Dannenberg, J.; Dapprich, S.; Daniels and 2009. Foresman, J.; Ortiz, J.; Cioslowski, J.; Fox, D. , Gaussian09 Revision D.01; Gaussian, Inc.: Wallingford, CT, .

9 C. Lee, W. Yang and R. G. Parr, *Phys. Rev. B*, 1988, **37**, 785–789.

10 P. C. Hariharan and J. A. Pople, *Mol. Phys.*, 1974, **27**, 209–214.
